# From misconceptions to empowerment: assessing health and genetic literacy on thalassemia among Tunisian secondary school students

**DOI:** 10.1186/s12889-025-24536-9

**Published:** 2025-10-08

**Authors:** Foued Maaoui, France Arboix-Calas, Ines Safra, Samia Menif, Imen Moumni

**Affiliations:** 1 Laboratory of Molecular and Cellular Hematology, Pasteur Institute of Tunis, Tunis, Tunisia; 2 ISEFC Bardo, Virtual University of Tunis, Tunis, Tunisia; 3 Faculty of Education, University of Montpellier, Montpellier, France

**Keywords:** Thalassemia, Health literacy, Genetic literacy, Common-Sense model, Tunisian youths, Primary prevention

## Abstract

**Context:**

Thalassemia, a prevalent hereditary hemoglobinopathy in Tunisia, poses significant public health challenges due to limited awareness, hindering prevention and management. This study evaluates health and genetic literacy among Tunisian secondary school students to inform targeted educational interventions.

**Method:**

We designed a questionnaire based on the Common-Sense Model of Self Regulation (CSM), a framework for understanding health threat responses, and Nutbeam’s health literacy framework, encompassing functional, interactive, and critical literacy across care, prevention, and health promotion. A cross-sectional study was conducted with 356 students (28.1% male, 71.9% female, aged 17–20) in public secondary schools. Participants reported their knowledge, affective, and behavioral attitudes toward thalassemia and its prevention.

**Results:**

Findings reveal low health literacy, with only 35% recognizing thalassemia’s hereditary nature and 46% mistakenly believing it is contagious. Significant differences were observed by study specialty (p < 0.05), with scientific students outperforming literary students. Over half of respondents failed to understand genetic transmission risks, and 33% saw no need to inform partners of carrier status. However, most expressed positive attitudes toward blood donation and supporting affected children.

**Conclusion:**

Early health education is critical for thalassemia prevention. This study, the first to assess Tunisian adolescents’ thalassemia literacy, highlights the need for curriculum reforms to enhance genetic and health literacy, empowering informed reproductive decisions and reducing disease burden through primary prevention.

**Supplementary Information:**

The online version contains supplementary material available at 10.1186/s12889-025-24536-9.

## Introduction

Thalassemia, a hereditary hemoglobinopathy, is a significant public health challenge in Tunisia, with prevalence rates of 7.38% for alpha-thalassemia and 2.21% for beta-thalassemia [1]. Caused by impaired hemoglobin chain synthesis, it leads to chronic hemolytic anemia, manifesting as fatigue, jaundice, and weakness. Treatment includes blood transfusions, iron chelation, and emerging gene therapies [2]– [3]. Patients face physical and psychosocial burdens, including stigma and reduced quality of life, worsened by societal misconceptions [4–9]. Despite its prevalence, low public awareness in Tunisia hinders prevention efforts like genetic counseling and premarital screening [10–14].

Limited health and genetic literacy among Tunisian adolescents exacerbates this issue, as early education is critical for informed reproductive decisions and primary prevention [15–18].

This study addresses this gap by evaluating secondary school students’ knowledge, perceptions, and attitudes toward thalassemia. The research questions are:

- What is the level of health and genetic literacy regarding thalassemia among Tunisian secondary school students, and how can these findings inform targeted health education interventions?
- How do affective and behavioral attitudes toward thalassemia and its prevention vary among students, and what implications do these have for designing psychosocial components in school curricula?
- How does study specialty (scientific vs. literary) influence health and genetic literacy, and what does this suggest for equitable curriculum reforms to support thalassemia prevention?

Health literacy, defined by the World Health Organization as the cognitive and social skills enabling individuals to access, understand, and use health information [16], is a cost-effective strategy for managing non-communicable diseases like thalassemia [15].

Genetic literacy, encompassing knowledge of inheritance and genetic risks, is equally vital for preventing hemoglobinopathies [17]– [18]. Research shows early genetic education fosters informed decision-making, reduces stigma, and supports participation in societal debates on genomic technologies [19–23].

For instance, Jennings (2004) frames genetic literacy as part of citizenship education, enabling personal and public engagement with genetics-related issues [24–26]. Countries like Cyprus have reduced thalassemia incidence through integrated genetic education and mandatory screening [27], unlike Tunisia, where such programs are absent.

In the Tunisian educational system, secondary school students (aged 15–20) are divided into scientific and literary tracks. Biology, including genetics, is primarily taught in scientific tracks, with sickle cell disease used to illustrate molecular genetics, but thalassemia is not covered. Literary students receive minimal genetics education, contributing to disparities in health literacy. Health education is embedded within biology and not offered as a standalone subject, limiting awareness of genetic disorders across all tracks. This curriculum gap highlights the need for reform to address hemoglobinopathies comprehensively.

Global studies underscore the importance of early education for hemoglobinopathy prevention. Alao et al. (2009) advocate for carrier screening and education among youth to halt disease spread [10], while Vasava (2009) found only 46% of Indian secondary school students were aware of hemoglobinopathies, emphasizing the need for early intervention [14].

In Tunisia, barriers such as limited access to genetic counseling, cultural stigma around hereditary conditions, and socioeconomic constraints perpetuate the disease burden, particularly in rural areas. These factors, combined with the absence of targeted prevention programs, necessitate health education that promotes both knowledge and psychosocial competencies.

This study fills a critical research gap by assessing Tunisian adolescents’ thalassemia literacy and attitudes, providing data to inform evidence-based curriculum reforms. By fostering health and genetic literacy from adolescence, Tunisia can empower future generations to engage in primary prevention, reducing the physical, psychosocial, and economic burden of thalassemia. The findings aim to support curriculum developers in designing a competency framework tailored to thalassemia prevention, encouraging informed decision-making and social engagement, such as blood donation and advocacy.

### Theoretical framework

#### Emotions play an important role in risk perception and decision-making in health

The cognitive perception of health risk is a family, educational, media and societal construct. It controls emotional intensity, such as fear of a risk factor or disease. This emotion in turn motivates decision-making and health behavior.

Consequently, People are driven to undertake preventative actions when they become aware of their vulnerability to danger [28]. – [29].

For Van der Pligt (1996) and Oh (2021), Those who perceive a larger danger of illness are more likely to pursue preventive measures [30]. – [31]. Turner (2012) and Tsoy (2021) confirm that emotions have the power to actively promote preventative actions in addition to influencing behavioral outcomes through risk perception [32]. – [33].

Lazarus (1994) and Archvadze (2021) consider that various attitudes and behaviors might be triggered by emotions. For instance, dread would make people seek out ways to solve problems or escape the circumstance they are afraid of [34]. – [35].

Emotions are widely used in health education [36]– [37]. For Toufan (2023), Artino (2012) and Gluck (2016), emotions affect cognition through four mechanisms: memory, cognitive resources, cognitive strategies, and motivation. These mechanisms can be effective in teaching self-regulation, reasoning, and academic success to students [38]. – [39]– [40].

Fear and empathy, for example, are known to be levers that induce a change in health behavior.

Fear appeals are widely used in health communications. Proponents of fear appeals, such as the Centers for Disease Control and Prevention (2014) [41], argue that they have a positive effect on attitudes, intentions, and behaviors [42]. 

The investigations by Karnaze et al. (2022) show an association between compassion and empathy with prosocial health behaviors and attitudes during the COVID-19 pandemic. They suggest that messages and interventions aimed at increasing compassion and empathy can promote public health behaviors during a pandemic [43]. 

Several theoretical models have incorporated coping information as a predictor of reactions to a threatening message. For instance, the Extended Parallel Process Model [44], which draws on the Drive Reduction Model [45], Leventhal’s Danger Control/Fear Control Framework (1970) [46], Protection Motivation Theory [47], and the Health Belief Model [48], predicts that responses to a perceived threat depend on the balance between perceived threat (severity and susceptibility) and perceived efficacy (self-efficacy and response efficacy). When perceived efficacy is high, individuals are motivated to protect themselves.

Consequently, thalassemia fear is an attribute of its perception, i.e. it influences the label given to the disease (hazardous or non-hazardous disease), its causes and consequences. It also influences controllability perception and preventive measures efficacity perception to be adopted in risk situations. No fear can lead to risk devaluation and inhibit decision-making, while excessive fear can generate an erroneous risk perception and a fatalistic conception “inevitable or insurmountable disease”.

For this reason, being aware of emotions and their effects on reproductive decisions and choices should be one of health education’s goals.

Leventhal et al. (1997) introduced the Common-Sense Model of self-regulation (CSM), which outlines the cognitive and emotional processes that impact the motivations for adopting adaptive actions [49]. 

This approach, which was initially created to take into consideration reactions to threats connected to health, can also be helpful for interventions meant to modify behavior in other domains where dangers are present [50].

In this study, we drew on CSM to design the thalassemia questionnaire items to assess knowledge, thalassemia representations, and affective and behavioral attitudes to hereditary risk (Table 1). The CSM includes behavioral and cognitive reactions in addition to the self-constructed sickness representation, and five dimensions are evaluable [51]. – [52]:

1. Identity: The designation or classification assigned to the illness and its accompanying symptoms. This establishes how serious the illness is thought to be.
2. Cause: any opinions, even if unfounded scientifically, regarding the origins of the illness.
3. Chronology: forecasts regarding the anticipated length of the illness.
4. Consequences: personal accounts of the illness effects and how they affect a person’s life.
5. Controllability: opinions regarding the preventability or treatment of the illness.

This study used identity, causes, consequences, and controllability -four CSM components- to assess knowledge and identify the affective variables influencing attitudes and behaviors. Table 1.

**Table 1:** Match between thalassemia representation, CSM dimensions and questionnaire items

Thalassemia representation	CSM dimensions	Objects	Items
Knowledge (Fig. 1)	Cause	Hereditary	A, H, M
Emotional determinant and behavioral attitudes (Table 5a et 5b)	Label	Hazardous/non-hazardous and Fear	G1
	Consequences	Physical Academic/Professional	B J
	Controllability	Communication Lifestyle choices Social commitment	I, K S, T, U, V L1, L2

### Health literacy and genetic literacy: two educational constructs for proactive health behaviors

Studies have been carried out on adult populations in several countries, provide evidence of a relationship between health literacy and health behaviors in adults [53]– [54]– [55]– [56], but few have focused on how this health literacy is constructed in schools with adolescents [57]. – [58].

Developing health literacy at an early age could bring lifelong benefits, whether in terms of disease prevention or management, which makes schools the most appropriate environment in which to introduce educational programs that build it.

It is increasingly recognized that adolescents should be encouraged to express themselves and be more autonomous in their health decisions [59]. – [60].

We believe that adolescents’ ability to assess hereditary risk should not be underestimated. Education has the potential to facilitate this self-assessment process through the development of reflective skills in youth, in an age-appropriate manner, it is an effective way for health literacy to translate into positive health behaviors [61]. – [62].

For this study, we consider health literacy (HL) to be an educational construct in conjoint with the scientific and academic learning targeted by the school. Health literacy is the set of cognitive and social skills that determine the motivation and ability of individuals to access, understand and use information about hemoglobinopathies in ways that promote and maintain good health.

In our questionnaire, we have devoted several items to the different forms of literacy: interactive, functional and critical, and their respective integrations with the four dimensions (access, comprehension, evaluation and application of information) and the three health domains (care, prevention and health promotion) as reported in the studies by Sorensen (2012) [63]. (Appendix 1)

Compared to genetic literacy (GL), thalassemia prevention requires a scientific understanding of the disease, i.e. the relationship between genetic information expression and hemoglobin disorders, and how this autosomal recessive disease is transmitted.

On a different register, thalassemia’s hereditary nature gives it a social identity as an “insurmountable” and “incurable” disease, as it relates to a genetic endogen.

In many countries, explanations such as divine punishment [64]– [65], bad luck and superstitious spells [66] are still dominant, leading to fatalistic attitudes on patients’ [67]– [68], as well as distrust or pity among their entourage [10].

Consequently, the genetics teaching at secondary school will have to fulfill not only its academic function, but also its social function as an agent of change against simplistic, reductionist and stigmatizing conceptions.

It’s up to genetic learning to overcome this social identity by developing a functional and humanistic genetic literacy. To assess the genetic literacy of our secondary school students, we asked them to answer several items on genetic counseling and prenatal diagnosis (Table 2).

**Table 2:** Match between the HL/GL dimensions applied to thalassemia and the objects and questionnaire items

	HL/GL dimensions	Objects	Items
Health literacy	Knowledge	Nature/Cause Symptoms Consequences Diagnosis Therapies Sampling techniques, screening, genetic counselling, MAP, etc.	A, H, M B J D C N, Q
	Skills	Emotional Informative Psychosocial Decision-making	G F I, K L1, L2
Genetic literacy	Knowledge	Dominance/Recessivity Autosomal transmission	O, P, R
	Skills	Problem solving (Interpretation/Assessment)	W, X, Y, Z, S, T, U, V

We believe that the data gathered from the study of youths’ knowledge and representations of thalassemia, and their genetic and health literacy levels will serve as a basis for devising a comprehensive education approach to primary prevention of thalassemia in Tunisia.

## Methodology

### Research design and participants

This is a quantitative study based on a cross-sectional survey. The study was conducted in two public secondary schools, Tajerouine School (Kef region, rural) and Nouvelle Medina School (Ben Arous region, urban), to capture diverse socio-economic and educational contexts.

These schools were selected due to their accessibility during the COVID-19 pandemic and willingness to participate, ensuring a mix of rural and urban student perspectives.

However, their selection may not fully represent the broader Tunisian education system due to regional variations in resources and health education exposure.

A multidisciplinary team made up of researchers from Molecular and Cellular Hematology Laboratory at the Pasteur Institute in Tunis (Tunisia) and Faculty of Education Montpellier (France) and verified the conceptual, semantic, and operational equivalency of the items during the design phase, in addition to the questions and references’ scientific relevance. The measures equivalency was checked by the team as well.

A closed questionnaire was developed for this study and used to gather data (Appendix2). Participants recruited from the scientific and literary classes, where the proportion of female students is twice that of male students, are included in our sample. (Table 3).

**Table 3:** Sample structure of secondary school students by gender, study specialty and district for an age group (17–20 years) (*n* = 356)

Total		Numbers 356	% Percentages 100
Gender	Male	100	28.1
	Female	256	71.9
Region and secondary school	Ben Arous/New Medina school	212	59,5
	Kef/Tajerouine school	144	40,4
Study specialty	Scientific	200	56.1
	Letters	156	43.8

The study involved 356 secondary school students, aged between 17 and 20, of which 68% were female and 32% were male. The gender distribution reflects the typical proportion of female students in scientific tracks in Tunisian secondary schools (approximately two-thirds female), but it may influence emotional variables, such as greater sensitivity to hereditary risks among females. Data collection occurred at the end of the first trimester of the school years 2021–2022 and 2022–2023 (October–December 2021 and October–December 2022). This period was chosen as it followed the completion of the human reproduction and genetics module in the Tunisian secondary school curriculum, ensuring students had relevant background knowledge. The timeline was determined in consultation with biology teachers to align with the curriculum schedule.

Participants were made aware of the guidelines regarding voluntary participation and anonymity before to filling out the questionnaire. They received assurances that stopping the questionnaire at any moment would have no negative effects. To prevent bias during interviewer administration, biology teachers were trained to use standardized scripted instructions and neutral prompting, avoiding leading questions. Anonymity was ensured through unique identifiers for responses, minimizing social desirability bias. The study was approved by the Pasteur Institute of Tunis’s ethical committee (CEBM: 2020/23/I/LR16IPT), with informed consent obtained from participants and their parents.

### Development, validation, and reliability of the questionnaire

To confirm the tool’s accuracy, we used a mixed validation method that included both experts and target users [69]– [70]– [71]– [72].

Herdman et al. (1998) propose a model for evaluating questionnaires, distinguishing six levels of equivalence: (1) conceptual (relevance of the tool to measure what it intends to measure), (2) item (appropriateness of the items to be measured), (3) semantic (appropriateness of the terms used), (4) operational (formulation of item layout and mode of use), (5) measurement equivalence (evidence of reliability and psychometric properties), and (6) functional equivalence (effective use across cultures) [70]. 

In the following section, we describe the conception, adaptation, and validation process of our questionnaires based on the model by Herdman (1998) and Tsai (2011) [70–72]. 

#### Questionnaire conception

Based on the HL literature, we developed a questionnaire on hemoglobinopathies that covers the Nutbeam framework (Nutbeam, 2000) [73], specifically functional and interactive HL (FHL and IHL) and critical HL (CHL) in three subdomains: critically appraising information, understanding health social determinants, and actions to address them, according to the Sorenson matrix (Sorenson, 2012) [63].

#### Questionnaire adaptation process

Two main phases can be distinguished in the adaptation process: the questionnaire evaluation phase and the pilot testing phase. In the questionnaire evaluation phase, we primarily addressed the conceptual, semantic, and operational equivalence of the items. This involved consulting experts, conducting cognitive interviews with secondary school students, and holding another experts’ meeting to integrate the results and finalize the questionnaire for the first pilot test.

**Fig. 1:**
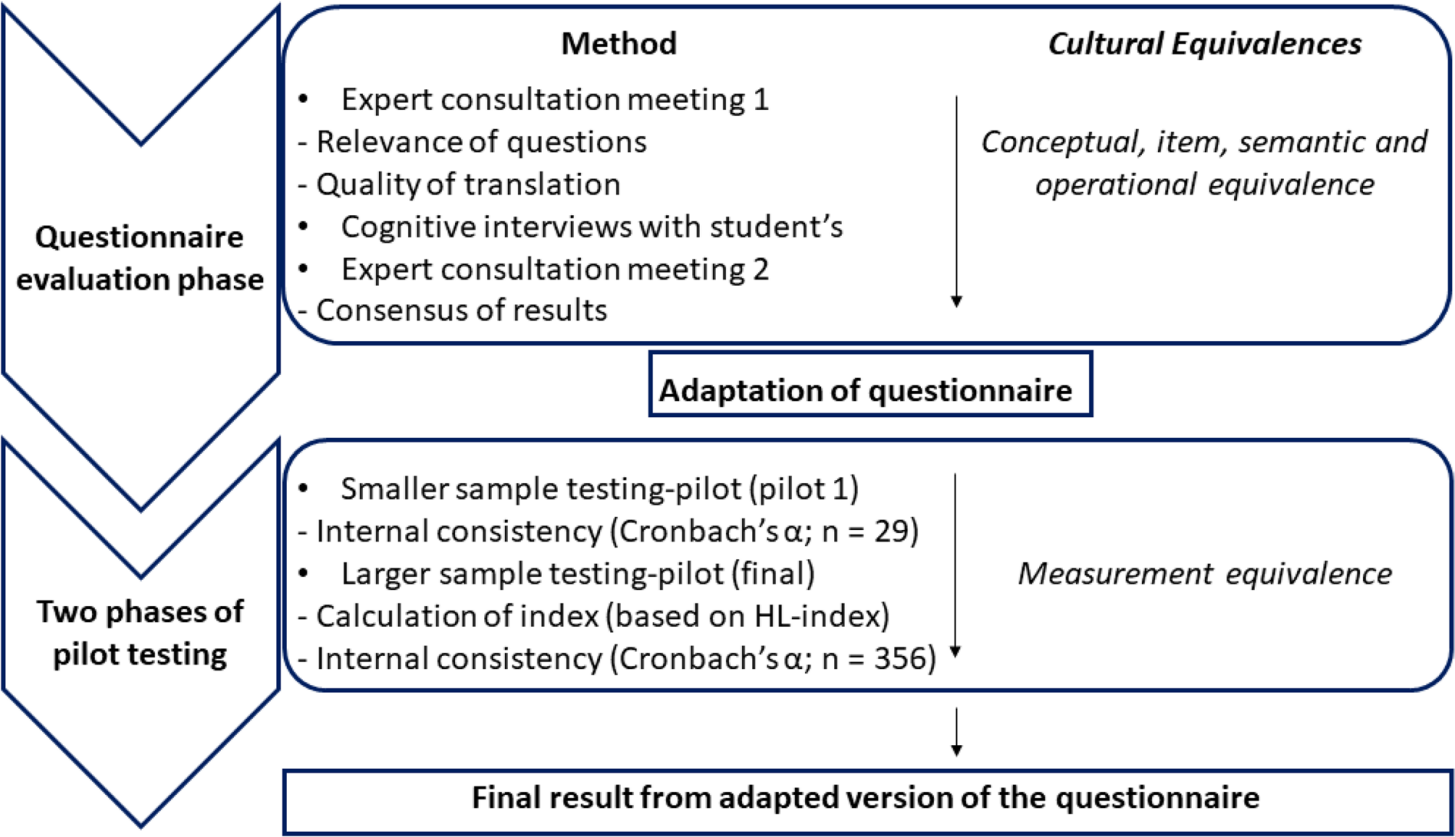
Validation flowchart for the HL thalassemia questionnaire

In the pilot testing phase, we assessed measurement equivalence by first conducting a small pilot test with a reduced number of participants, followed by a larger investigation with a greater number of students. The process is illustrated in Fig. 1.

#### Questionnaire evaluation phase

Experts’ consultation and interviews: Experts were consulted on specific criteria, such as expertise in hematology, hemoglobinopathies, education, research methodology and biostatistics. (Table 4).

**Table 4:** Characteristics of respondents in the questionnaire / evaluation phase

Characteristic and Respondent	n	Explanation/Remark
Expertise		
Hematology (Pr. MD)	2	Pasteur Institute of Tunis, Tunisia
Haemoglobinopathies (PhD)	1	
Health education (PhD)	1	Faculty of Education Montpellier, France
Biology Didatic (PhD. Student)	1	Virtual University of Tunis, Tunisia
Research Methodology (PhD)	4	
Biostatistics	1	
Students for in-depth interview		
Boy	2	In two public secondary school, scientific grades Terminal
Girls	4	

Students were recruited for cognitive interviews through a purposive selection process based on their academic streams and levels, ages (17–19). This group exclusively represented youths enrolled in public schools located in Greater Tunis who had received instruction in human genetics. Contact with adolescents facilitated the translation, cultural appropriateness, terms and wording, structure, response alternatives, question order, administration instructions, layout, and identification of possible typographical errors [70–74]. 

At the meeting, experts were invited to give their opinions: “What do you think this questionnaire is trying to measure? How does it relate to the primary prevention of thalassemia? Is it justified to ask adolescents these questions?”

Cognitive interviews with youths were conducted after the first expert meeting to check the items’ validity based on interactions and original objectives.

Students were encouraged to think aloud, i.e., to report on the mental processes they used to produce an answer [75]. 

The interviewer asked respondents to define terms or concepts (e.g., “What does the term screening mean to you?”), to check whether they had studied certain concepts (e.g., “Have you already completed the course in human genetics?”), or to interpret certain proposed questions or situations (e.g., “Can you tell me in your own words what this question means to you?”).

The interviews were designed to provide a better understanding of the items’ conceptual, semantic, and operational equivalence. The students were also asked about the questionnaire’s structure and administration. The average interview lasted between 50 and 60 min.

The questionnaire validation process involved iterative revisions after each interview, followed by testing for internal consistency (Table 5).

**Table 5:** Summary of the questionnaire validation process

Validation Step	Main Objective	Methods Used	Results/Decisions
1. Iterative Pre-testing	Adapt questions based on participant feedback	Questions were revised after each interview and the updated version was tested in the next interview	Continuous improvement of question wording
2. Internal Consistency	Examine internal coherence of the items	Revised questionnaires were tested to assess internal consistency	Results shared with experts for further evaluation
3. Conceptual Equivalence	Ensure the tool measures the intended construct	Experts reviewed questionnaire items before the meeting to assess content validity	Validation of content covering thalassemia, symptoms, diagnosis, screening, and counseling
4. Item Equivalence	Ensure items are appropriate in length and function	Health literacy items were rated as favorable (+) or unfavorable (–) regarding relevance, representativeness, and clarity	Discrepant ratings were discussed until expert consensus was reached
5. Semantic Equivalence	Ensure terminology is understandable to secondary school students	Bilingual (French-Arabic) questionnaire created; evaluated on a 3-point scale (1 = different, 2 = almost identical, 3 = identical); cognitive interviews with 15 students	Mean score = 2.8 (SD = 0.3); reformulation favored over exclusion; consensus achieved
6. Cultural Relevance	Ensure alignment with the linguistic and educational context	Selection of students familiar with scientific vocabulary in both languages due to French-medium biology curriculum	Terminology adapted to reflect both linguistic realities
7. Operational Equivalence	Ensure appropriateness of format, order, response options, and administration	Expert discussions covered format, instructions, administration mode; interviewer-administered format preferred; need to train teachers for standardized delivery	Questionnaire format validated; self-administration discouraged; standardized administration recommended
8. Measurement Equivalence	Evaluate psychometric properties of the adapted tool	Two pilot tests conducted; assessed construct validity, internal consistency, and floor/ceiling effects using small and large student samples	Adapted questionnaire found to be statistically reliable

Expert consultations ensured conceptual equivalence, confirming that the tool accurately measured key aspects of thalassemia, including symptoms, diagnosis, screening, and genetic counseling. Item equivalence was assessed by rating each item’s relevance, clarity, and representativeness, with discrepancies resolved through expert consensus.

To ensure semantic equivalence, a bilingual (French-Arabic) version was created and tested using a 3-point scale (1 = different meaning in each version, 2 = almost identical 362 meaning in both versions, and 3 = identical meaning in both versions) in cognitive interviews [76] with 15 students, achieving a mean score of 2.8 (SD = 0.3). Cultural relevance was ensured by selecting students familiar with scientific terminology in both languages. Items with different meanings were revised rather than excluded to preserve psychometric integrity. Operational equivalence was addressed by adapting the questionnaire’s format, instructions, and administration mode, with a preference for interviewer administration and training recommended for teachers. Finally, measurement equivalence was verified through pilot testing in small and large samples, assessing construct validity, internal consistency, and floor/ceiling effects, confirming the questionnaire’s statistical reliability.

### Sampling strategy

In this study, we evaluate the effectiveness of the current school curriculum for the prevention of thalassemia. The inclusion and exclusion criteria used to select the sample are being enrolled in a public high school, being in the 3rd or 4th year of secondary education, and having obtained written parental consent to respond to the questionnaire.

The investigation was conducted in a pandemic context related to COVID-19, so we opted for convenience sampling. Out of about ten randomly selected schools in different regions of Tunisia, only four agreed to host us for the study. We were able to obtain parental consent from participants at only three sites; the first served as our initial pilot test.

In the other two, we surveyed students whose parents had given prior consent and because the principals and teachers agreed to host us in their classrooms.

The sample size (*n* = 356) was determined using Slovin’s formula (n = N/(1 + Ne²)) with a 5% margin of error, ensuring statistical robustness. Convenience sampling was employed due to COVID-19 restrictions, with only two schools (Tajerouine and Nouvelle Medina) participating based on accessibility and consent. The use of convenience sampling, necessitated by logistical constraints and the need for parental consent, is a limitation that may affect the generalizability of the findings. While these schools represent urban and rural contexts, the sample may not fully capture Tunisia’s diverse educational landscape, as discussed in the limitations.

For the first pilot test, a class of 29 secondary school students were randomly recruited in Ibn-Mandhour school. These youths had never participated in a previous survey. A biology teacher administered the questionnaire in the presence of the interviewer.

We then conducted a large-scale cross-sectional investigation: 356 adolescents aged 17 to 20 completed the questionnaire. This age group corresponds to the 3rd and 4th years of secondary school. Students were recruited by random sampling from two public secondary schools: Tajerouine School (Kef region) and Nouvelle Medina School (Ben Arous region).

### Data analysis

To analyze the data, we utilized SPSS, v.25.0, statistical software for the social sciences.

First, we performed normality analyses for items located in the second section of the questionnaire (i.e. level of thalassemia knowledge), the third (i.e. ability to assess inheritance and the usefulness of genetic counselling and prenatal diagnosis) and the fourth (affective and behavioral attitudes towards thalassemia). We next assessed each study topic using parametric tests, which were based on the outcomes of our normality assessments.

We used Pearson’s chi-square test and Fisher’s exact test as inferential statistical tools to analyze the categorical data, in particular to examine whether there was a significant difference in knowledge scores and proactive attitudes towards thalassemia according to study specialty (scientific or literary).

The hypothesis that the difference in the level of literacy in health and genetics depends on the study specialty was statistically tested. The lower the significance value (Sig. < 0.05), the less likely it is that the two variables are independent. If the significance value is very low, this means that the two variables are indeed linked.

All items were analyzed using descriptive statistics (i.e. mean values, standard deviations, frequencies and percentages).

To assess the reliability and internal consistency of the measurement scales, we used three psychometric tests. A Cronbach’s alpha, a Guttman split-half reliability coefficient and a Spearman-Brown coefficient.

Firstly, we used Cronbach’s alpha to measure the internal consistency of our scale. This test measures the reliability or internal consistency of a group of questions. Cronbach’s alpha values range from 0 to 1, with higher values indicating greater internal consistency. In this study, internal consistency was high, with Cronbach’s alpha values between 0.8 and 0.9.

Secondly, we employed the Split-Half method, which involves randomly dividing the items into two groups with an equal number of items. We then calculated the partial score for each group for all participants and determined the correlation between the two partial scores.

Thirdly, we used the Spearman-Brown formula to estimate the reliability of the complete test based on the correlation from the Split-Half method. A higher correlation coefficient indicates better internal consistency.

Floor and ceiling effects are present if 15% or more of the questionnaire respondents answer with the lowest or highest possible score [77]. There were no missing items, as the interviewer could help explain any items that adolescents did not directly understand. A normal distribution of data was found in both tests, with no floor or ceiling effects.

## Results

### Questionnaire reliability and validity

The data collection questionnaire consisted of four parts:

- The first part contained a personal data sheet with questions on gender, age, residence, school level and study specialty.
- The second part was composed of four items dealing with the origin, symptoms, diagnosis and therapy, with several propositions to be ticked. The Pearson chi-square is significant for the study specialty (Sig < 0.05).
- The third part included a ten-item trichotomous scale (yes/no/no idea) with (α Cronbach: 0.813, M: 2.17, SD: 0.750), a Guttman Split-Half reliability coefficient of 0.712 and a Spearman-Brown coefficient of 0.713.

Six items were used to assess participants’ level of knowledge about the genetic transmission of thalassemia. They were asked to tick “Yes”, “No” or “No idea” for each statement.

Avec α Cronbach: 0.705, M: 2.13, SD: 0.853, a Guttman split-half reliability coefficient of 0.713 and a Spearman-Brown coefficient of 0.731.

Four items were proposed to assess the judgement abilities of secondary school students confronted with four hereditary risk situations. They were asked to tick “No risk”, “Obvious risk” or “No idea” (α Cronbach: 0.825, M: 2.31, SD: 0.678).

- The fourth part was composed of nine items, one item for the affective component aimed at self-evaluating their fear of thalassemia. Seven levels ranging from “Not afraid” to “Very afraid” were given to the students (α: 0.751, M: 1.91, SD: 0.667) with a Guttman Split-Half reliability coefficient and a Spearman-Brown coefficient of 0.751.

Then four items that assess participants’ attitudes towards thalassemia on a 4-point Likert-type scale (1 = strongly disagree, 2 = somewhat disagree, 3 = somewhat agree and 4 = strongly agree). This behavioral component is divided into three subsections: communication (two items; α = 0.606, M: 2.45, SD: 1.153), and social engagement (two items; α = 0.907).

Lifestyle choices with 4 items to assess the perceived usefulness of genetic counselling and prenatal diagnosis depending on the situation proposed, they had to tick “Not useful”, “Useful” or “No idea” (α Cronbach: 0.731, M: 2.11, SD: 0.669), with a Guttman Split-Half reliability coefficient and a Spearman-Brown coefficient of 0.785.

### Secondary school students’ perception of thalassemia

#### Place of thalassemia among the diseases most feared by secondary school students

**Fig. 2:**
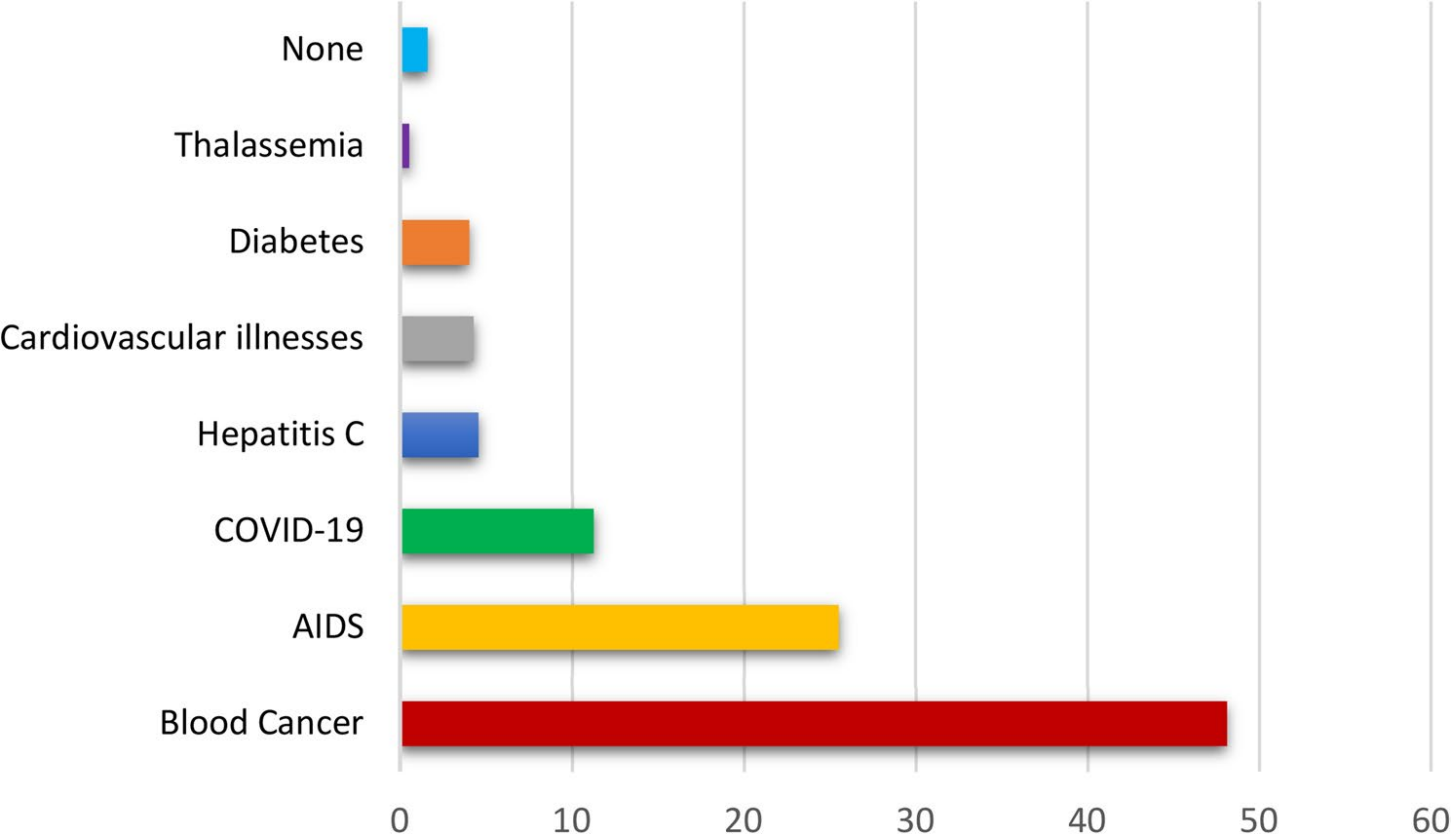
Perception of thalassemia dangerousness compared to other diseases

We proposed a list of seven diseases: blood cancer, infectious diseases, metabolic, cardiovascular diseases and hereditary diseases. We then asked our students to rank the most dangerous diseases, they ranked thalassemia last (Fig. 2).

They do not consider thalassemia to be a dangerous disease because they are unaware of its physical and psychosocial consequences.

### Some representations of thalassemia

#### Contagiousness

A significant number of respondents were uncertain about the hereditary origin of thalassemia, with 46% agreeing with statement H: “Some forms of thalassemia are contagious” (Table 6).

**Table 6:** Representations of “contagiousness” and “disability” in relation to thalassemia

		Strongly disagree	Somewhat disagree	Somewhat agree	Strongly agree	*N*	Mean	SD
Contagiousness								
H. Some forms of thalassemia are contagious.	f	88	104	72	92	356	2.53	1.124
	%	24.7	29.2	20.2	25.8			
Disability								
J. When you have thalassemia, you are not able to study or work like a healthy person.	f	85	130	100	41	356	2.73	0.953
	%	23.9	36.5	28.1	11.5			

####  Disability

More than half (60.4%) of respondents did not perceive thalassemia as a disabling disease or as an obstacle to study or work. Nearly (40%) agreed with statement J: “When you have thalassemia, you are not able to study or work like a healthy person.” (Table 6).

SD: Standard Deviation; f: frequency; %: percentage of responses from secondary school students.

### Health literacy: secondary school students’ level of knowledge about thalassemia

This subsection presents findings on students’ general knowledge of thalassemia, including disease symptoms, diagnosis and therapies. Questionnaire items A, B, C and D respectively assess students’ knowledge about nature, symptoms, therapies and diagnosis of thalassemia:

#### The nature of thalassemia

**Fig. 3:**
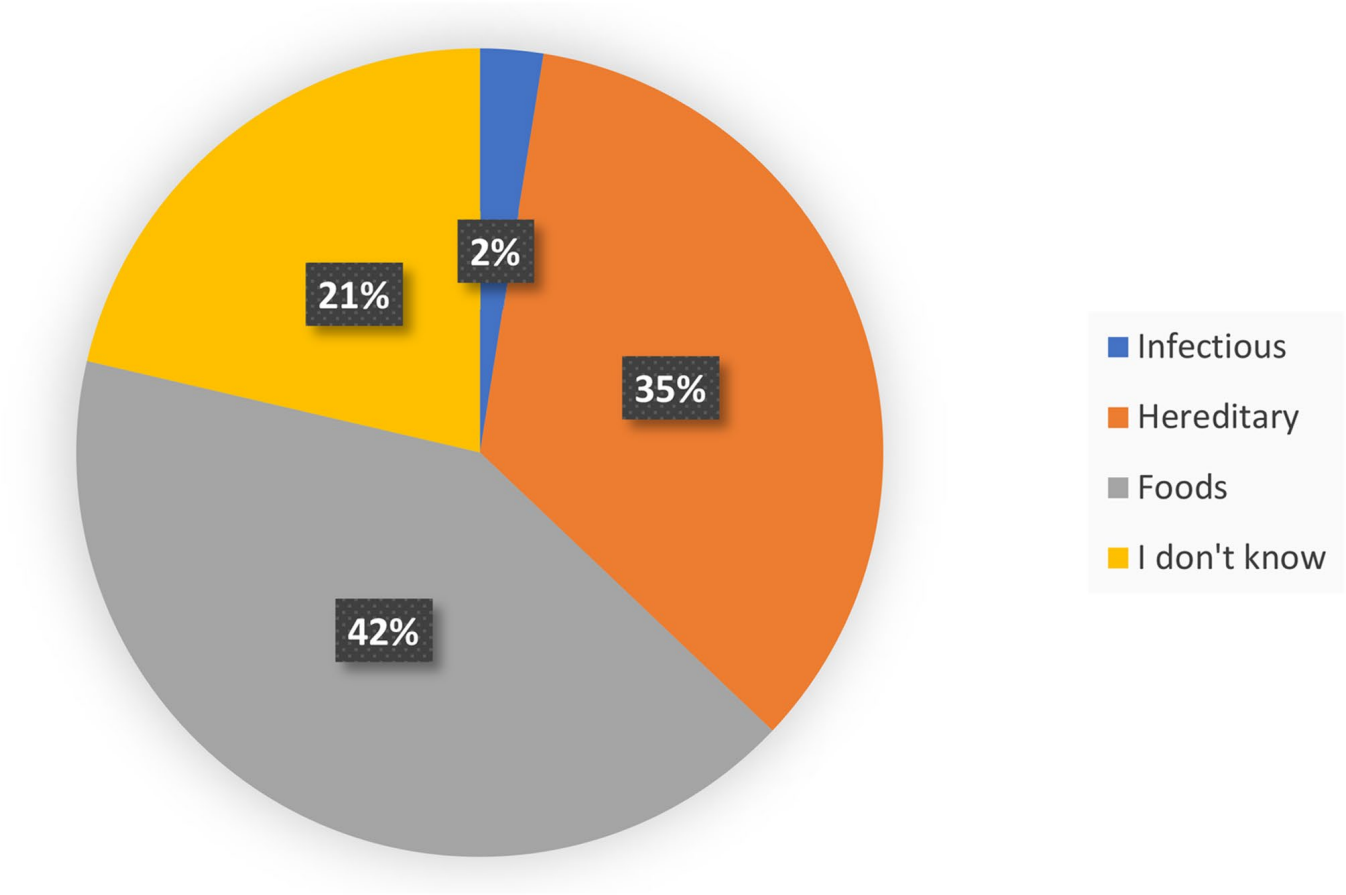
Percentage of responses on the origin of thalassemia

We asked the question (A): “Thalassemia is a disease: (1) infectious, (2) hereditary, (3) dietary, (4) I don’t know” to our secondary school students. (42%) of respondents opted for a dietary, hereditary (35%) or infectious (2%) origin. While (21%) said they did not know the nature of thalassemia (Fig. 3).

There was a significant difference between the experimental sciences section and the letters section (Table 7), with 41.4% (3Sc) and 57.5% (4sc) respectively aware of thalassemia hereditary nature, compared with only 11.8% (3 L) and 16.9% (4 L) with (Pearson’s χ2 = 63.91, Sig < 0.001).

**Table 7:** Percentage of responses on the origin of thalassemia by study specialty

	3rd Sc	Vs	3rd L	4th Sc	Vs	4th L
Infectious (%)	1,2		5,9	0		4,2
Hereditary (%)	41,4		11,8	57,5		16,9
Food (%)	42,5		52,9	28,3		47,9
Don’t know (%)	14,9		29,4	14,2		31,0

3rd year Experimental Sciences (TN)/11th grade (Junior Year of High School) (US).

3rd year Letters (TN)/11th grade (Junior Year of High School) (US).

4th Experimental Sciences (TN)/12th grade (Senior Year of High School) (US).

4th Letters (TN)/12th grade/Senior Year of High School (US).

#### Thalassemia symptoms

**Fig. 4:**
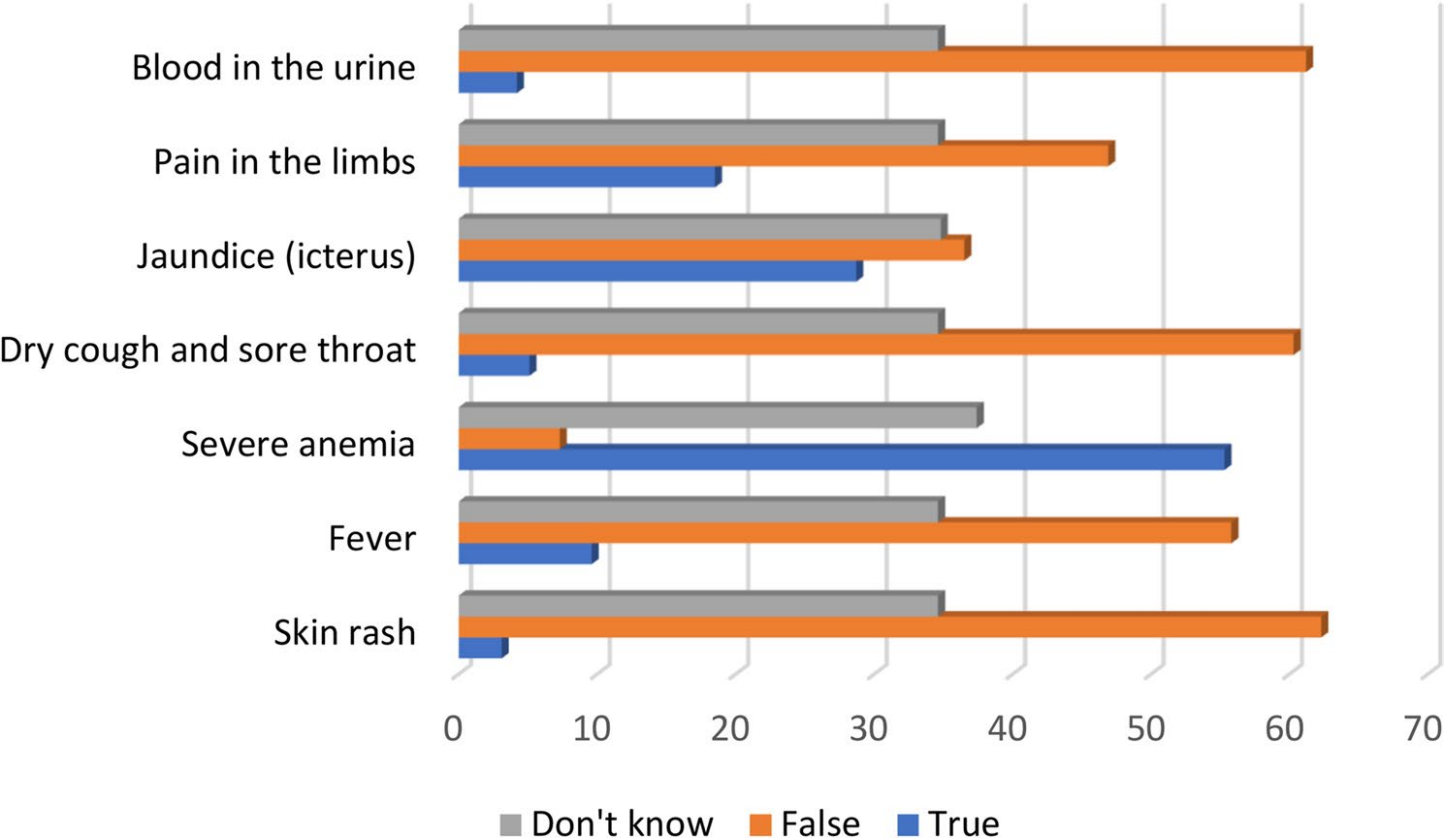
Percentage of responses on the symptoms of thalassemia

We asked our students question (B) about the symptoms of thalassemia, with seven choices to be confirmed or refuted: (1) blood in the urine, (2) pain in the limbs, (3) jaundice, (4) dry cough and sore throat, (5) severe anemia, (6) fever, (7) skin rash.

(55.3%), (28.7%) and (18.5%) respectively identified severe anemia, jaundice and limb pain as symptoms of thalassemia. More than a third of participants said they did not know the symptoms of this haemoglobinopathy (Fig. 4).

Knowledge of the symptoms is different according to study specialty (Pearson’s χ2 = 83.05, Sig < 0.001), with respectively 78.16% (3Sc) and 71.68% (4Sc) of the scientific section recognizing anemia as a symptom of thalassemia, compared with 29.41% (3 L) and 32.39% (4 L) of the literary section (Table 8).

**Table 8:** Identification of anemia symptoms according to study section

	3rd Sc	Vs	3rd L	4th Sc	Vs	4th L
True (%)	78,16		29,41	71,68		32,39
False (%)	6,9		5,88	9,73		5,63
Don’t know (%)	14,94		64,7	18,58		61,97

3rd year Experimental Sciences (TN)/11th grade (Junior Year of High School) (US).

3rd year Letters (TN)/11th grade (Junior Year of High School) (US).

4th Experimental Sciences (TN)/12th grade (Senior Year of High School) (US).

4th Letters (TN)/12th grade/Senior Year of High School (US).

#### Thalassemia diagnosis

**Fig. 5:**
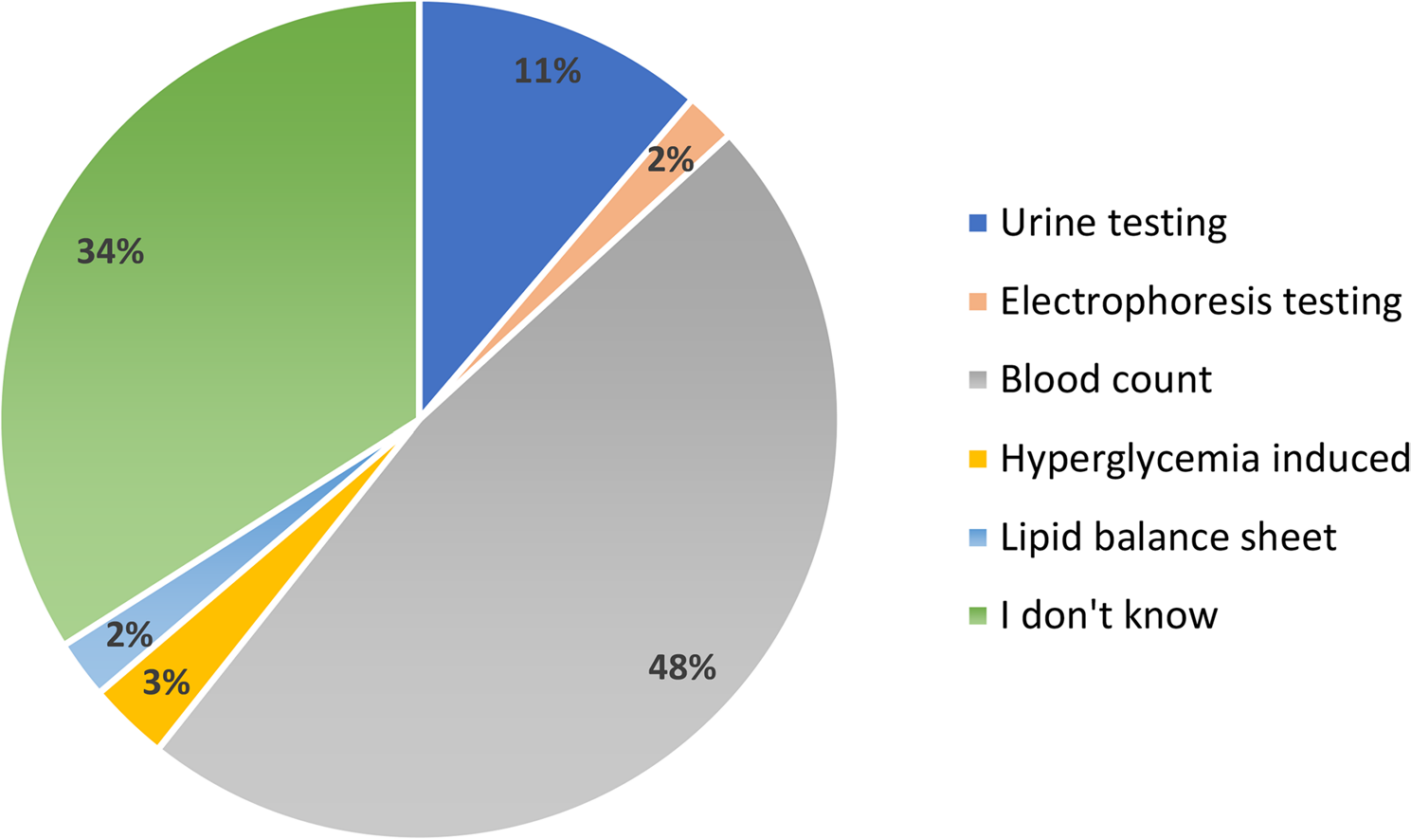
Percentage of responses on the diagnostic techniques for thalassemia

To answer question (D), participants were asked to tick the diagnostic tools for haemoglobinopathies from a list of five options: 48% of secondary school students identified blood count but 98% did not recognize electrophoresis as a diagnostic option. 34% said they had no answer to this question (Fig. 5).

#### Thalassemia treatment

**Fig. 6:**
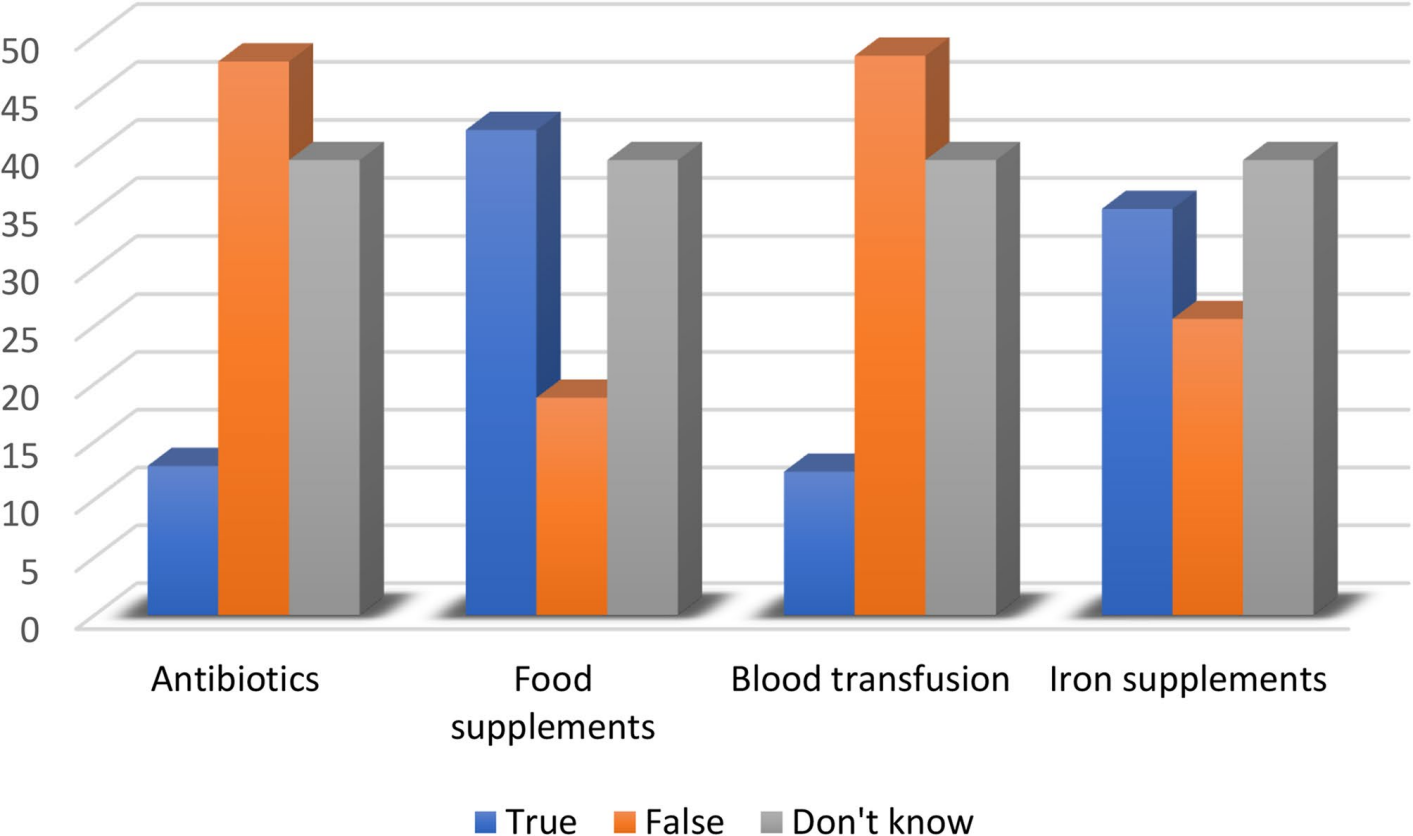
Percentage of responses on the treatment options for thalassemia

From a list of four therapies proposed in question (C), most participants opted for food supplements (41.9%) and iron supplements (35.1%), while (12.9%) chose antibiotics and only (12.4%) identified blood transfusions (Fig. 6).

Data from Tunisian secondary school students reveal significant misconceptions about thalassemia, reflecting the absence of structured health education in the national curriculum and the influence of sociocultural factors. Only 35% of students correctly identified thalassemia as a hereditary disease, while 42% mistakenly attributed it to dietary or infectious causes, and 21% were unaware of its origin.

Additionally, 46% erroneously agreed that some forms of thalassemia are contagious, indicating a fundamental misunderstanding of genetic disorders (Table 6). Regarding symptoms, 55.3% recognized severe anemia, but only 28.7% and 18.5% identified jaundice and limb pain, respectively, with many associating unrelated symptoms like dry cough or fever.

Diagnostic knowledge was equally limited: 48% selected blood count as a relevant test, but 98% failed to identify hemoglobin electrophoresis, the standard diagnostic method for hemoglobinopathies, and 34% provided no answer.

Treatment misconceptions were prevalent, with 41.9% and 35.1% selecting inappropriate food or iron supplements, respectively, while only 12.4% correctly identified blood transfusion as the primary therapy. These findings highlight a critical lack of accurate health literacy, posing risks of perpetuating harmful misconceptions and delaying proper care.

The Tunisian education system, which rarely addresses the issue of genetic inheritance or chronic disease management for non-scientific sections, contributes significantly to these shortcomings. Sociocultural factors, including stigma around hereditary conditions, taboos surrounding consanguinity, and fatalistic interpretations of illness, further reinforce misinformation, often derived from hearsay or social media.

A notable disparity exists between scientific and literary students (Table 9): scientific students (*n* = 198) scored a mean of 72.3% (SD = 8.1) on knowledge assessments, significantly higher than literary students’ 58.7% (SD = 9.4; *n* = 158; *p* < 0.001).

**Table 9:** Knowledge scores by study specialty

Study Specialty	Mean Score (%)	Standard Deviation	*p*-value
Scientific (*n* = 198)	72.3	8.1	*p* < 0.001
Literary (*n* = 158)	58.7	9.4	

This gap reflects the scientific curriculum’s focus on genetic concepts, with 85% of scientific students correctly identifying thalassemia as genetic compared to 62% of literary students. Rural literary students, 42% of whom linked thalassemia to social shame, are particularly affected by cultural stigma, exacerbating their knowledge deficits.

These findings underscore the urgent need for comprehensive health education and culturally sensitive interventions to address misconceptions and enhance thalassemia awareness across all student groups.

#### Literacy in human genetics applied to thalassemia

This subsection details students’ understanding of genetic concepts related to thalassemia, such as inheritance patterns.

To assess genetic literacy as applied to thalassemia, we proposed four questions for respondents to confirm or refute (Table 10):

- The first question (M) concerned the “contagiousness of haemoglobinopathies”. Half of the participants (54.8%) answered correctly, while the other half said that you can catch haemoglobinopathies like flu (15.7%) or had no idea (29.5%).
- The second question (N) assessed the notion of hereditary risk controllability: “Can a sick man avoid having sick children? only 29.8% of participants answered correctly, while two-thirds answered falsely (28.7%) or had no answer (41.5%).
- The third question (Q), like the second, assessed the controllability of haemoglobinopathies: “Can a woman with thalassemia avoid having sick children? only 34.8% of the students gave the correct answer, while two-thirds gave a wrong answer (19.9%) or no answer (45.2%).
- The fourth question (R), “Could a husband who is heterozygous for thalassemia and his wife who is homozygous for thalassemia have a sick daughter? was answered correctly by 41.6% of students, whereas 58.4% answered falsely (6.7%) or had no answer (51.7%).

**Table 10:** Assessment of genetic literacy applied to thalassemia

	Yes (%)	No (%)	No idea (%)
R. Could a husband who is heterozygous for thalassemia and his wife who is homozygous for thalassemia have a sick daughter?	41,6	6,7	51,7
Q. Can a woman with thalassemia avoid having sick children?	34,8	19,9	45,2
N. Can an ill man avoid having sick children?	29,8	28,7	41,5
M. Catching haemoglobinopathy is like catching the flu?	15,7	54,8	29,5

**Fig. 7:**
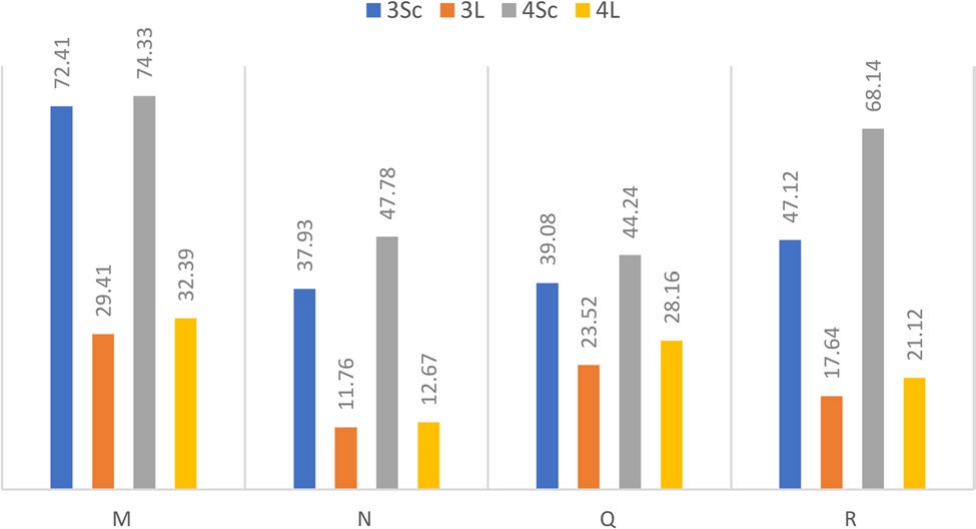
Genetic literacy applied to thalassemia: rate of correct answers according to study specialty

A comparison of the rate of correct answers for the four questions (Fig. 7) reveals a significant difference depending on whether the student is studying experimental sciences or humanities: M (Pearson’s χ2 = 71.95, Sig < 0.001); N (Pearson’s χ2 = 50.06, Sig < 0.001); Q (Pearson’s χ2 = 41.06, Sig < 0.001); R (Pearson’s χ2 = 70.47, Sig < 0.001).

The knowledge acquired in molecular, Mendelian and human genetics seems to favour the experimental sciences section at the expense of the humanities section, where the level of genetic literacy is lower.

Despite this advantage, the success rate for the experimental science section was less than 50% for the questions (N and Q). These questions concern the nature and transmission of haemoglobinopathy, as well as its controllability (avoidance). It seems clear that the skills developed in genetics by the secondary school students (3Sc and 4Sc) were not fully used to answer these questions, which can be explained by their ignorance of thalassemia and its mode of transmission.

#### Assessing the genetic risk of thalassemia

**Fig. 8:**
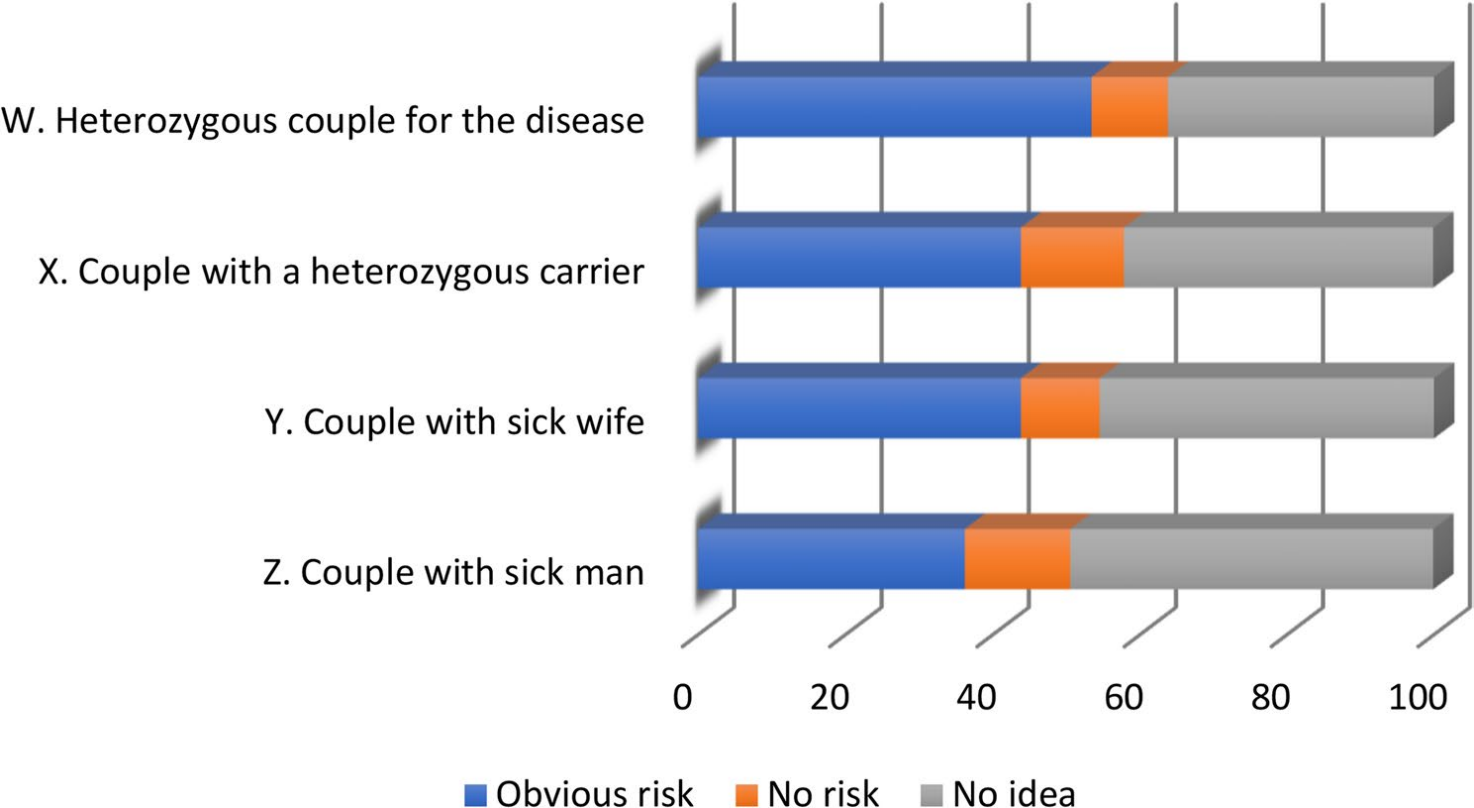
Assessing the risk of thalassemia genetic transmission

Thalassemia is an autosomal recessive disease. Four situations were proposed to respondents to assess the risk of having children with symptomatic thalassemia (Fig. 8):

- First situation (W): “a couple with two partners heterozygous for haemoglobinopathy”, half of the respondents considered that there was an obvious risk of having sick children, while the other half answered falsely as they saw no risk (10.4%) or had no idea (36.2%).
- Second situation (X): “a couple with a heterozygous carrier for haemoglobinopathy”, (43.8%) falsely believe there is a risk, while only (14%) believe there is no risk, and (42.1%) say they have no idea.
- Third situation (Y): “a couple with a sick woman”, knowing that we did not mention whether the man is a healthy carrier or not, there is a risk that the future children will be sick, (43.8%) consider that there is a risk while (10.7%) say there is not, (45.5%) have no idea.
- Fourth situation (Z): “a couple with a sick man”, knowing that we did not mention whether the woman is a healthy carrier or not, there is a risk that the future children will be sick, (36%) feel that there is a risk while (14.3%) do not see the risk, (49.4%) have no idea.

**Fig. 9:**
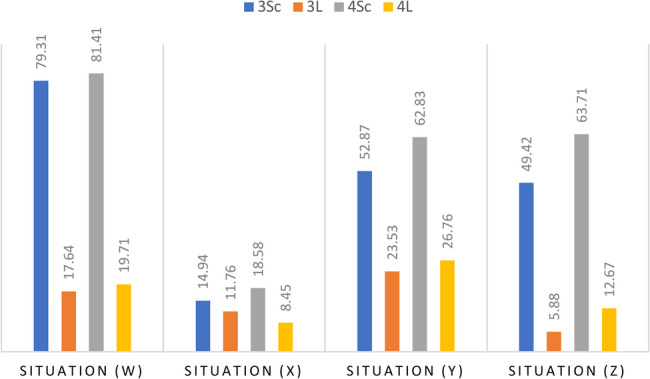
Assessing the risk of thalassemia genetic transmission by study speciality

The rate of correct answers to the risk situations (Fig. 9) also varied according to the study specialty, in favor of the experimental sciences section: W (Pearson’s χ2 = 46.99, Sig < 0.001); X (Pearson’s χ2 = 43.03, Sig < 0.001); Y (Pearson’s χ2 = 72.83, Sig < 0.001); Z (Pearson’s χ2 = 56.17, Sig < 0.001).

Learning in formal genetics (3Sc) and human genetics (4Sc) made it easier to understand the technical terms and assess the risk for each situation.

However, the success scores for situations (X, Y and Z) are average to low, which confirms the previous conclusion. Secondary school students in the experimental sciences section have good genetic literacy but low health literacy. They are capable of understanding and solving human genetics problems, but they are specifically unaware of thalassemia and its mode of transmission. The didactic analysis showed the absence of thalassemia in the life sciences curriculum taught at Tunisian secondary schools.

### Emotional and Behavioral Attitudes

This subsection explores students’ attitudes toward genetic counseling and prenatal diagnosis, highlighting emotional and behavioral trends.

#### Emotional attitudes of Tunisian secondary school students towards thalassemia

To estimate levels of thalassemia fear, we asked respondents to “Rate your level of fear of thalassemia on an increasing scale from 1 to 7”.

**Fig. 10:**
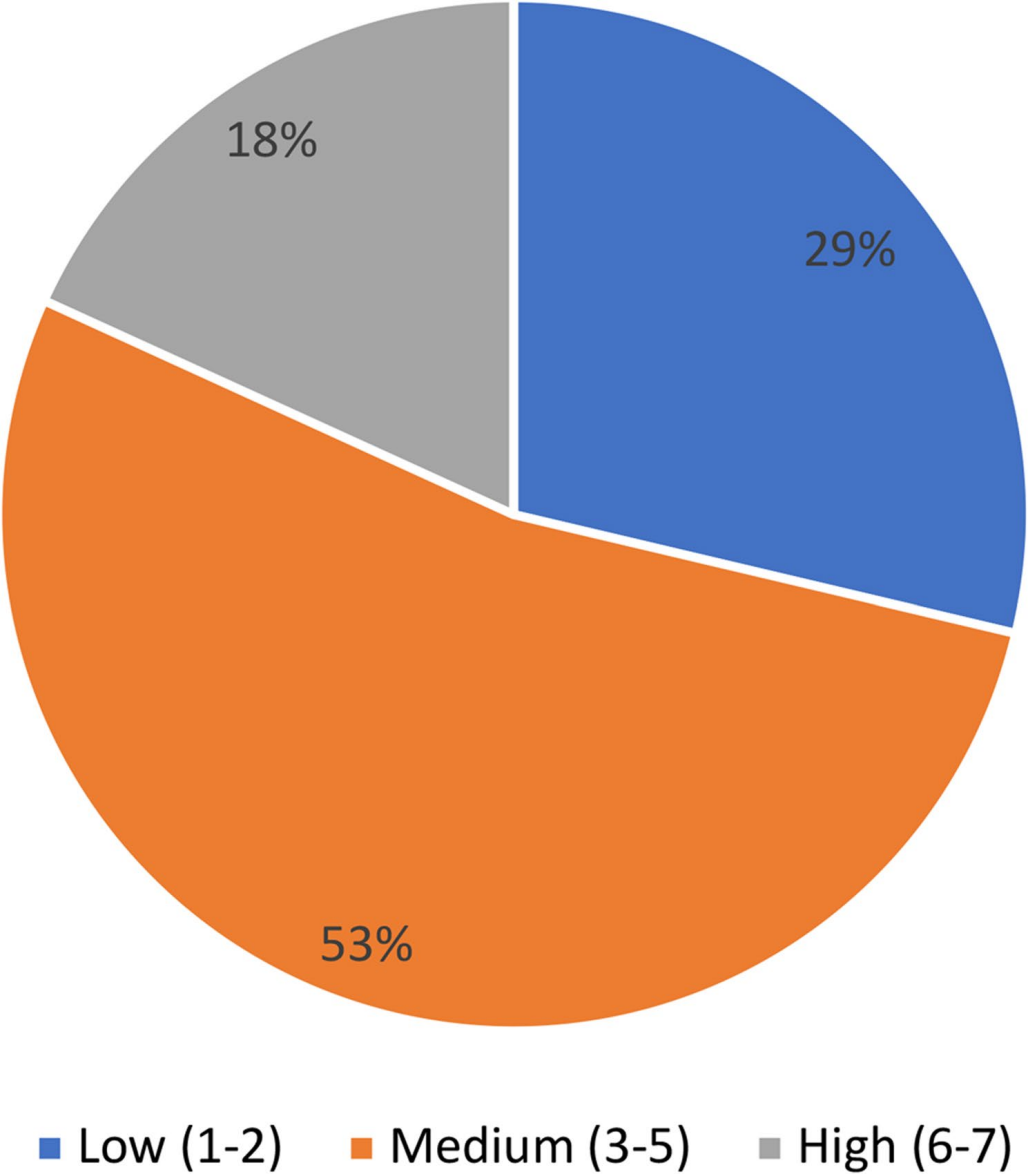
Self-reported level of thalassemia fear

The seven levels of fear were categorized as: (1–2) ‘low level’, (3–5) ‘medium level’ and (6–7) ‘high level’. The results show that (29%) said they were “not afraid”, (53%) “moderately afraid” and only 18% “very afraid”. (Fig. 10)

There was no significant difference according to study specialty, and the content taught did not seem to have an emotional impact on students’ perceptions, which can be explained by the absence of thalassemia in the Tunisian school curriculum.

#### Behavioral attitudes of Tunisian secondary school students towards thalassemia

##### Lifestyle choices

**Fig. 11:**
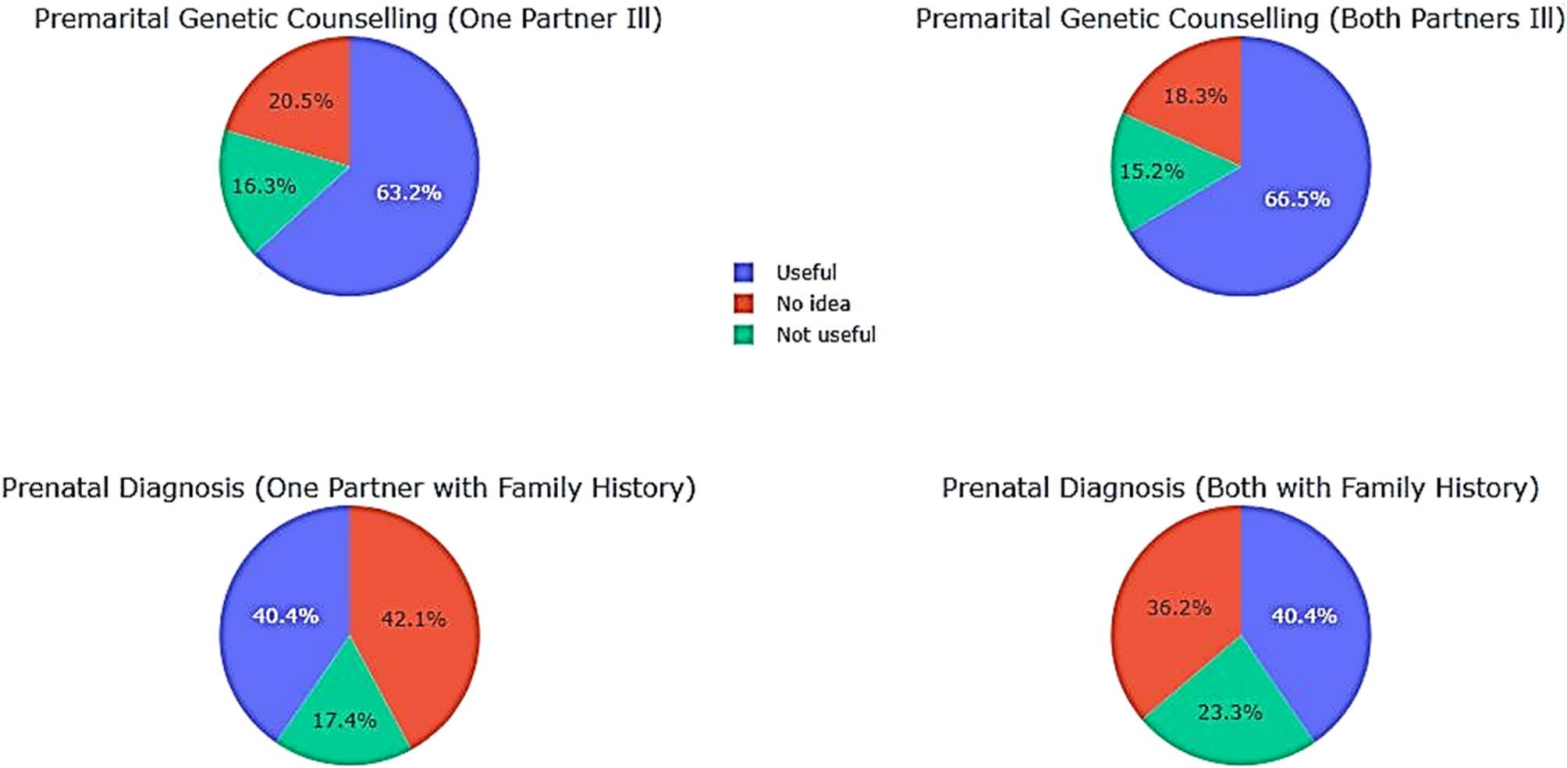
Behavioral attitudes towards thalassemia: genetic counseling and diagnosis

According to Table 8a, more than 50% of secondary school students seemed to agree with the items in the choice and lifestyle sub-section (S and T) of the behavioral dimension (M = 2.03). (Table 11)Nearly 65% of participants considered genetic counselling ‘useful’ before marriage when one or both partners were ill. 15.7% did not see it as useful and 19.4% had no idea (Fig. 11).

The results are less clear-cut with prenatal diagnosis, with only 40% believing it to be “useful” when the couple are healthy but one or both have a family antecedent of thalassemia.

**Table 11:** Participants’ level of agreement with statements on the behavioral dimension of attitudes towards genetic counselling and prenatal diagnosis of thalassemia

Lifestyle choices							
Faced with the idea of transmitting thalassemia to future children, what do you think of:		Useful	Not useful	No Idea	N	Mean	SD
Genetic counselling							
S. Premarital genetic counselling when one of the partners is ill.	f	225	58	73	356	2.04	0.606
	%	63.2	16.3	20.5			
T. Premarital genetic counselling when both partners are ill.	f	237	54	65	356	2.03	0.578
	%	66.6	15.2	18.3			
Prenatal diagnosis							
U. prenatal diagnosis when the couple is healthy but the husband has disease in his family.	f	144	62	150	356	2.25	0.732
	%	40.4	17.4	42.1			
V. prenatal diagnosis when the couple are healthy but have a family history of the disease.	f	144	83	129	356	2.13	0.762
	%	40.4	23.3	36.2			

SD: Standard Deviation; f: frequency; %: percentage of responses from secondary school students

##### Communication and social commitment

For the “communication” sub-section (Table 12), the responses were divided for item **I** (M = 2.74) between those who agreed (48.9%) and those who disagreed (51.1%) with the statement: “When you have thalassemia, it’s better to hide it from those around you (friends, etc.) in order to live a normal life”.

For item **K** of the communication sub-section (M = 2.16), (63.2%) of the participants agreed with the statement “If you have thalassemia, you must inform your partner of your illness before marriage”, whereas (36.8%) disagreed, probably because it could harm their chances of getting married.

**Fig. 12:**
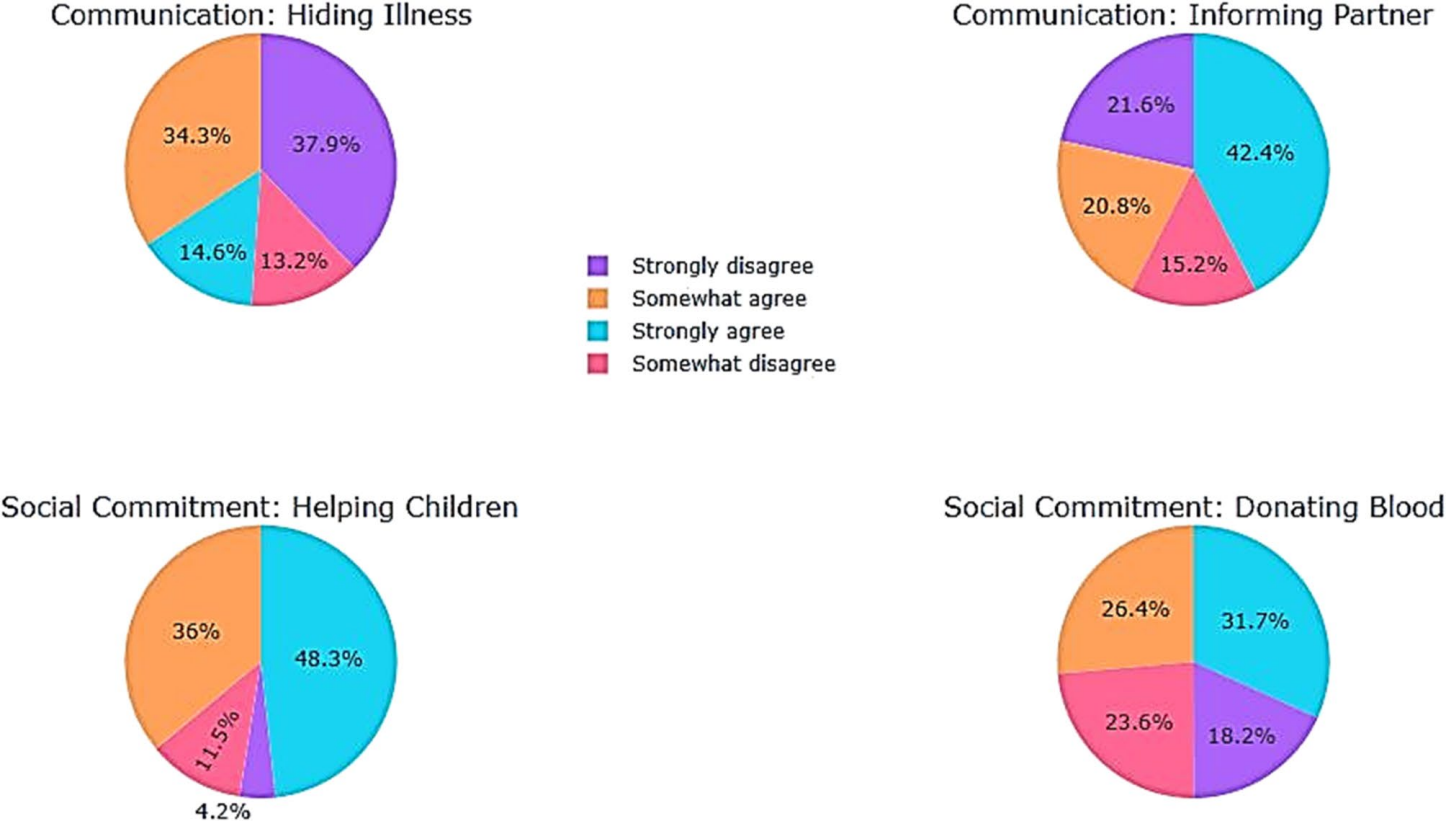
Behavioral attitudes towards thalassemia: communication and social commitment

Most respondents agreed with the “social commitment” sub-section (M = 2.99), with (84.3%) and (58.1%) respectively for the items (Fig. 12):

“L1. The idea of contracting the disease motivates me to help the children affected” and “L2. The idea of contracting the disease motivates me to donate blood”.

**Table 12:** Participants’ level of agreement with statements on the behavioral dimension of attitudes towards thalassemia/Communication and social commitment

		Strongly disagree	Somewhat disagree	Somewhat agree	Strongly agree	*N*	Mean	SD
Communication								
I. When you have thalassemia, it’s preferable to hide your illness from those around you (friends, etc.) so that you can live a normally.	f	135	47	122	52	356	2.74	1.115
	%	37.9	13.2	34.3	14.6			
K. If you have thalassemia, you must inform your partner of your condition before getting married.	f	77	54	74	151	356	2.16	1.191
	%	21.6	15.2	20.8	42.4			
Engagement social								
I. The thought of contracting the disease motivates me to help the children affected.	f	15	41	128	172	356	3.28	0.830
	%	4.2	11.5	36	48.3			
J. The thought of contracting the disease motivates me to donate blood.	f	65	84	94	113	356	2.71	0.985
	%	18.2	23.6	26.4	31.7			

SD: Standard Deviation; f: frequency; %: percentage of responses from secondary school students

## Discussion

### Cultural Context and the Limits of Genetic Counseling

Premarital screening and genetic counseling are widely recognized as essential strategies for identifying at-risk couples and enabling informed decisions regarding marriage. However, in Arab-Muslim cultures, marriage involves complex emotional, familial, and religious considerations. Even when couples are informed of their genetic risk, many still choose to marry due to cultural values and fear of social stigma associated with breaking engagements or annulments [78, 79]. In some contexts, prenatal diagnosis and therapeutic abortion are available, yet these options are often avoided due to religious prohibitions or moral hesitation. These findings highlight the crucial need to strengthen health literacy related to hemoglobinopathies, including thalassemia, in ways that are compatible with the sociocultural landscape [27]. Without culturally informed education, prevention strategies may have limited reach and effectiveness [27, 78, 79].

### Curricular Gaps and Misconceptions in Secondary Education

An analysis of Tunisia’s biology curriculum for public secondary schools reveals that thalassemia is not mentioned, and sickle cell disease appears only marginally, and solely as an academic concept. Core aspects such as prevalence, severity, symptoms, psychosocial impact, and available therapies are omitted. Consequently, students lack the foundational knowledge required for informed health-related decisions. Survey findings confirm widespread misconceptions: many students confuse thalassemia with iron-deficiency anemia or infectious diseases, indicating severe gaps in health literacy. These misunderstandings are more pronounced among students in literary tracks, reflecting inequitable curricular exposure to genetics and chronic disease education.

This educational disparity is not unique to Tunisia. Similar findings have been reported in Saudi Arabia [80] and Bangladesh, where students from scientific backgrounds demonstrated significantly higher knowledge levels compared to their peers from literary disciplines [81]. Studies consistently demonstrate that targeted education enhances students’ acceptance of prevention strategies and promotes informed behaviors [80–85]. For example, Iran’s integration of mandatory premarital screening and school-based education has led to a significant reduction in thalassemia incidence [85]. Indonesian studies also show improved knowledge and attitudes toward premarital screening among Muslim couples following tailored educational interventions [86]– [87].

### Health and Genetic Literacy: Current Status and Implications

The current status of health and genetic literacy among Tunisian adolescents reveals a profound gap between the demands of contemporary public health challenges and the educational preparation offered by the school system. Despite the increasing relevance of hereditary diseases such as thalassemia in Tunisia and across the Mediterranean region, students, particularly those outside the scientific track, have limited access to accurate, contextualized, and actionable information about these conditions. The findings of this study indicate that even when students demonstrate a basic awareness of genetic inheritance, their understanding remains superficial, fragmented, and often clouded by persistent misconceptions.

In many cases, students confuse thalassemia with iron-deficiency anemia, underestimate the severity and chronic nature of the disease, or are unaware of its prevalence and genetic transmission. Such confusion is not merely a reflection of individual ignorance; it points to deeper systemic deficiencies in how health and genetics are addressed in the educational landscape. The lack of a structured curriculum on health literacy [88] and the marginal treatment of genetic diseases within biology courses contribute to a passive and disengaged approach to personal and familial health. Moreover, the absence of emotional and civic framing around hereditary health risks limits students’ ability to relate biomedical knowledge to their own lives and social responsibilities.

The implications of this low level of genetic literacy are significant. Without a clear understanding of how genetic diseases are inherited and how they affect individuals and families, adolescents are unlikely to recognize the importance of preventive measures such as premarital screening or genetic counseling. This absence of understanding hinders the development of health-promoting attitudes and behaviors, both at the individual and community levels. In a context where consanguineous marriages remain culturally accepted and often preferred, the lack of genetic awareness may perpetuate avoidable health burdens and intergenerational suffering.

Furthermore, the study shows that even students in the scientific track, who have relatively more exposure to genetics, struggle to integrate what they learn into meaningful frameworks for action. The content is often presented in a purely technical or abstract manner, without connection to the sociocultural realities or emotional experiences of the students. This disconnection inhibits the development of what could be termed functional genetic literacy: the capacity not only to comprehend biomedical information but also to use it in making informed decisions that are emotionally grounded and socially responsible.

Addressing these shortcomings requires a redefinition of health and genetic literacy as more than the acquisition of factual knowledge [88]. It must be understood as a multidimensional competence that includes cognitive, emotional, ethical, and civic dimensions. A literate individual in this sense is not only capable of understanding genetic information but also of assessing its personal and societal implications, communicating effectively about health risks, and acting in ways that promote collective well-being.

### Functional Literacy in Human Genetics

Functional literacy in human genetics refers to the capacity to understand, interpret, and apply genetic information to real-life contexts such as health risk assessment, reproductive decision-making, and disease prevention. The results of this study reveal critical deficiencies in this domain among Tunisian secondary school students. Nearly half of the respondents did not know that thalassemia is a hereditary condition. A significant portion of students, regardless of academic track, held erroneous beliefs, including the idea that thalassemia is either infectious or caused by nutritional deficiencies. Even among students in the scientific stream, where some exposure to genetics is expected, the understanding of fundamental mechanisms, such as autosomal recessive inheritance, was inconsistent and fragmented. Only a small minority demonstrated the ability to accurately assess genetic risk within premarital or reproductive scenarios, highlighting a concerning lack of both durable and applicable knowledge.

This situation is even more pronounced among students in the literary track, who receive little to no instruction in genetics as part of their formal education. These students struggled significantly with questions related to genetic mechanisms, risk perception, and disease prevention. The absence of genetics education in this track reveals a profound equity gap: it systematically excludes a substantial portion of the youth population from engaging meaningfully with health information that is vital for their personal and familial well-being. This exclusion perpetuates social and health inequalities, particularly in a context where hereditary diseases like thalassemia are highly prevalent and closely linked to reproductive choices.

The complexity of genetic concepts compounds the problem. Genetics relies heavily on specialized, abstract terminology, such as “heterozygote” or “autosomal”, that is rarely encountered in everyday language. As several studies have emphasized [89–92], mastering such terminology is indispensable for understanding core biological processes. However, this linguistic barrier creates an additional layer of exclusion for students with limited scientific exposure or weak proficiency in academic language. The role of language in learning becomes particularly salient when viewed through the lens of Vygotski’s socioconstructivist theory [93], which posits that conceptual development is inextricably linked to mastery of the language tools used to mediate knowledge. From this perspective, students cannot develop robust scientific understanding without opportunities to engage with, and appropriate, the specific discourse of genetics.

Considering these findings, it is clear that improving functional literacy in human genetics requires much more than increasing content coverage. It demands pedagogical approaches that scaffold the acquisition of technical vocabulary, contextualize genetic knowledge in meaningful scenarios, and provide equitable learning opportunities across all academic tracks. Without such reform, a large segment of the student population will remain ill-equipped to make informed health decisions, thereby limiting the effectiveness of any public health strategy aimed at preventing hereditary conditions like thalassemia.

Addressing functional genetic literacy must be framed as both an educational and public health imperative. Equipping all students with the ability to understand and act upon genetic information is not only a matter of cognitive development but also of health justice, especially in societies facing a high burden of genetic diseases and entrenched disparities in access to scientific education.

### Emotional and Behavioral Attitudes Toward Thalassemia

Surveyed students did not perceive thalassemia as a serious disease. Many ranked it below cancer, cardiovascular disease, or diabetes in terms of perceived danger. Fear of being affected was low, especially among male students. Female students expressed more concern, suggesting gendered differences in health perception. However, the overall absence of fear signals a lack of disease awareness, not courage or negligence.

Misinformation was also evident in attitudes toward contagion and life limitations. A substantial proportion believed thalassemia was contagious and did not interfere with academic or professional success. Such misconceptions diminish students’ perceived urgency to engage in preventive actions.

On the behavioral side, while half of the students supported premarital counseling, only 40% found prenatal diagnosis useful. Concerns about needle procedures, risk to the fetus, and fear of abortion decisions may explain this hesitancy. In terms of communication, many agreed that hiding illness was preferable, a belief likely rooted in fear of stigma. Students observed that society, school, and the workplace are often unkind to the chronically ill, a finding consistent with other studies documenting the stigma surrounding hemoglobinopathies [7, 8, 94, 95].

Only half of respondents would inform their partners of their carrier status, highlighting social and familial pressures. Nevertheless, in the domain of social engagement, most students expressed willingness to support thalassemia-affected children and agreed on the value of blood donation, even without understanding its role in treatment. These attitudes can be leveraged through awareness programs.

### Structural Determinants: Socioeconomic, Cultural, and Religious Barriers

Efforts to improve health literacy must contend with broader structural and cultural barriers. In Tunisian and other Arab-Muslim societies, thalassemia prevention strategies, such as genetic counseling or partner screening, challenge prevailing beliefs about fate (“maktoub”), divine will, and the sanctity of marriage.

Macrosocial inequalities exacerbate these challenges. In Tunisia, the absence of a national carrier screening policy and the limited availability of specialized care centers reflect a broader trend of prioritizing communicable diseases over hereditary disorders, which affect politically and economically marginalized populations. The epidemiological hierarchy favors diseases that pose a public health threat over those that cause chronic personal suffering. Without international funding or public pressure, non-transmissible diseases like thalassemia remain neglected.

Bio-social inequalities are also evident. Women bear a disproportionate burden, both as carriers and caregivers. In many Arab-Muslim cultures, women are often blamed for their children’s genetic conditions [66, 96]. This leads to silence, stigma, and isolation. Fear of judgment or abandonment often prevents mothers from seeking timely care. Children with thalassemia experience school discrimination due to their condition being poorly understood. These findings echo studies across the Middle East that highlight gendered vulnerability and silence surrounding genetic conditions.

### Curricular Implications and a Path Forward

The findings of this study point to critical shortcomings in the current Tunisian educational curriculum, particularly in its ability to prepare students, regardless of academic track, for active engagement in health prevention. The biology curriculum, as it stands, offers limited, decontextualized information on hereditary diseases, often disconnected from real-world concerns and emotional relevance. This disconnect is particularly pronounced in the literary and technical tracks, where students have little or no exposure to genetic topics. As a result, large segments of the adolescent population are denied access to the knowledge and critical thinking skills necessary to understand, assess, and respond to hereditary health risks such as thalassemia.

The absence of a structured and inclusive health education component in the national curriculum further exacerbates these inequities. This structural void reinforces patterns of social and scientific exclusion, whereby only students in science-focused streams have partial access to biomedical information, while others remain uninformed or misinformed. Such asymmetry in knowledge access deepens existing disparities and weakens collective health preparedness. Therefore, reform is urgently needed to integrate thalassemia-related content into a cross-cutting health education program, accessible to all students, regardless of academic specialization.

A curriculum reform guided by the principles of health literacy would not only improve scientific knowledge but also foster personal relevance and emotional engagement, two essential conditions for behavioral change. In this regard, the Common-Sense Model (CSM) of illness representation provides a valuable theoretical framework. By addressing students’ lay perceptions and misconceptions about health threats, early educational interventions can reshape cognitive representations and encourage proactive health behaviors. Applying the CSM to school-based health education allows educators to engage with students’ intuitive beliefs and help them develop more accurate, evidence-based understandings of genetic risks, including thalassemia [97].

Such an approach calls for the development of interdisciplinary modules that blend biology, ethics, civic education, and psychosocial perspectives. These modules should emphasize not only the biomedical dimensions of thalassemia but also its emotional, familial, and social implications. Introducing these topics in a way that speaks to students’ lived experiences can bridge the gap between abstract content and real-life decision-making. This is particularly relevant in a context like Tunisia, where cultural, religious, and familial values significantly influence marriage choices and reproductive health behaviors.

A path forward would involve phased implementation of curricular innovations, beginning with the integration of pilot modules in selected schools. These modules should be evaluated for pedagogical impact and adapted accordingly before national scaling. Simultaneously, teacher training initiatives must be developed to equip educators with both content knowledge and participatory pedagogical techniques. Collaboration with public health institutions, NGOs, and community leaders will be essential to ensure that educational reforms are scientifically grounded and socially relevant.

Curricular transformation must be conceived not as an isolated educational endeavor, but as a public health imperative. By embedding genetic literacy and preventive thinking within the educational system, Tunisia can take a decisive step toward empowering its youth to make informed, ethical, and culturally sensitive health decisions [63, 88]. Such reform will serve not only the goal of thalassemia prevention but also the broader objective of strengthening civic responsibility and health resilience among future generations.

### Recommendations for Policy and Practice

The findings of this study strongly advocate for the formulation and implementation of a comprehensive, long-term strategy to address the educational and public health gaps in thalassemia prevention among Tunisian youth. A coordinated national response, grounded in evidence and adapted to the sociocultural context, is necessary to build the genetic and health literacy required for meaningful primary prevention. This response must operate on multiple time scales, short, medium, and long term, each involving specific actions, institutional collaboration, and sustained political commitment.

In the short term, the priority should be to build national capacity for delivering accurate, relevant, and culturally sensitive education on thalassemia and genetic risk. This begins with targeted training for biology and health education teachers at the secondary school level. Educators must be equipped not only with scientific knowledge about hemoglobinopathies but also with pedagogical tools and strategies for engaging adolescents from diverse academic and social backgrounds. Teacher training should integrate content on genetics, hereditary transmission, premarital screening, and sociocultural perceptions of disease, while fostering inclusive classroom dialogue to challenge stigma. Simultaneously, awareness campaigns tailored to youth should be launched at the national level, using digital platforms, school-based events, and media to disseminate accessible, age-appropriate information on thalassemia. These campaigns should be developed in partnership with health professionals, educators, and civil society organizations to ensure credibility and reach.

Over the medium term, Tunisia should aim to integrate a dedicated thalassemia education module into the official secondary school curriculum. This module should be interdisciplinary in nature and delivered not only in scientific streams but also in literary and technical tracks, thus ensuring equitable access to knowledge regardless of academic orientation.

The educational content must go beyond the biological aspects of the disease and include psychosocial, cultural, and ethical considerations. The module should emphasize the hereditary nature of thalassemia, the importance of premarital screening, the challenges faced by affected individuals, and the potential of preventive measures. Classroom activities may involve case studies, group discussions, role-playing scenarios, and peer education strategies to foster both cognitive and emotional engagement. Institutional collaboration between the Ministries of Education and Health will be essential to support curriculum design, teacher recruitment and training, and school-level implementation. A national monitoring and evaluation framework should be established to track the effectiveness of the new curriculum in terms of knowledge acquisition, attitude change, and behavioral intentions among students.

In the long term, the goal should be to institutionalize a national thalassemia prevention policy that includes both educational and biomedical components. This policy would mandate premarital genetic counseling and carrier screening as part of routine reproductive health services, while also embedding genetic education within the broader public health strategy. To support this objective, specialized centers for genetic counseling and hemoglobinopathy care should be established or reinforced across the country, particularly in underserved regions. These centers would not only provide diagnostic and therapeutic services but also serve as hubs for community education and support.

Public communication campaigns should continue to evolve, addressing stigma, promoting solidarity with affected families, and normalizing conversations around genetic risk. Religious and community leaders should be engaged to ensure that prevention messages are compatible with prevailing moral and cultural frameworks, particularly with respect to marriage, reproduction, and divine will.

Crucially, all these actions must be informed by ongoing research and data collection. This includes assessing students’ evolving levels of genetic literacy, tracking the uptake of premarital screening, and evaluating the social impact of education programs. Regular feedback from teachers, students, parents, and health professionals should be integrated into program revisions, ensuring that thalassemia education remains relevant, effective, and inclusive. Collaborative efforts with regional and international partners may provide technical expertise, financial support, and opportunities for comparative evaluation.

Overall, these policy recommendations emphasize the importance of a multi-sectoral, equity-focused, and culturally grounded approach to thalassemia prevention. By investing in health and genetic literacy through the education system, Tunisia can equip its youth with the knowledge and agency to make informed reproductive decisions, reduce hereditary disease burden, and foster a public health culture that is inclusive, proactive, and responsive to emerging challenges.

### Pilot Thalassemia Education Module: Concept and Evaluation

In light of the study’s findings and the critical need to enhance genetic and health literacy among Tunisian adolescents, we propose the development and experimental implementation of a pilot thalassemia education module in selected secondary schools. This module is envisioned as both a pedagogical and public health intervention, aiming to increase students’ understanding of thalassemia, its hereditary transmission, and the importance of preventive behaviors such as premarital screening and informed reproductive decision-making.

The conceptual framework guiding the module’s design is rooted in health literacy theory, which emphasizes not only the acquisition of factual knowledge but also the development of critical thinking, risk perception, and decision-making skills in health-related contexts. In addition, educational psychology principles, particularly those related to active learning, socio-constructivist engagement, and emotional involvement, will inform the instructional strategies used. The module is thus intended to promote not only cognitive comprehension but also emotional and ethical reflection, enabling students to make sense of thalassemia not simply as a genetic disorder but as a socially situated condition that affects individuals, families, and communities.

The content of the module will be interdisciplinary and adapted to the Tunisian curricular context. It will cover basic genetic concepts relevant to hemoglobinopathies, the epidemiological profile of thalassemia in Tunisia and the Mediterranean region, the clinical manifestations and psychosocial consequences of the disease, and the available preventive strategies. A particular emphasis will be placed on understanding autosomal recessive inheritance patterns, the implications of consanguineous marriage, and the social and cultural representations of genetic diseases. The module will also encourage reflection on students’ own values, beliefs, and future aspirations with regard to marriage and health, fostering a sense of personal relevance and agency.

In terms of pedagogy, the pilot module will prioritize active learning methods that encourage student participation and peer interaction. These may include scenario-based discussions, group debates, role-playing exercises, problem-solving tasks, and the analysis of testimonial materials from individuals living with thalassemia. The goal is to create a learning environment where students feel empowered to ask questions, challenge misconceptions, and engage critically with the content. The use of digital tools, audiovisual resources, and culturally adapted materials will further enhance accessibility and engagement.

The evaluation of the pilot module will adopt a mixed-methods approach to assess both its feasibility and its educational impact. Quantitative data will be collected through pre- and post-intervention questionnaires measuring students’ knowledge, attitudes, and intentions regarding thalassemia and genetic screening. These instruments will be developed based on validated health literacy and genetic literacy scales, adapted to the local context. Qualitative data will be gathered through focus group discussions and interviews with students, teachers, and school administrators, aiming to capture perceptions of the module’s relevance, clarity, cultural appropriateness, and emotional impact. Teachers will also be asked to document their experiences and challenges in delivering the module, providing essential feedback for future adjustments.

The implementation will be conducted in collaboration with the Ministry of Education, regional educational authorities, and local health professionals, ensuring that the module is both scientifically accurate and pedagogically sound. Schools will be selected based on their geographical, sociocultural, and academic diversity, allowing the evaluation to capture a range of learning contexts and identify potential barriers to scale-up.

The ultimate aim of this pilot initiative is to generate empirical evidence that can inform national curriculum reform and guide the integration of genetic health education into the broader framework of adolescent health promotion. If proven effective, the module could serve as a model for other health-related topics requiring sensitive, interdisciplinary, and youth-centered approaches. It would also position Tunisia as a regional pioneer in the educational response to hereditary diseases, aligning with global public health strategies promoting health literacy and disease prevention from an early age.

### Future Directions and Research Priorities

Building upon the insights gained from this study, several future directions emerge to deepen the understanding of health literacy and to optimize educational strategies for thalassemia prevention among adolescents. First, it is essential to expand the empirical research base through longitudinal studies that assess the long-term impact of school-based genetic education interventions. While short-term improvements in knowledge and attitudes are valuable, they do not necessarily translate into sustained behavioral changes.

Future studies should therefore investigate whether early exposure to health education influences young people’s reproductive decisions, including their willingness to undergo premarital screening or seek genetic counseling in adulthood.

Another priority involves exploring the differential effects of educational interventions across various sociocultural, regional, and socioeconomic contexts in Tunisia. The country exhibits considerable heterogeneity in terms of health service access, educational quality, and cultural attitudes toward genetic disorders. Understanding how these contextual factors shape adolescents’ reception of health information, and their decision-making processes will be crucial for designing equitable and culturally appropriate interventions. Comparative studies involving rural and urban populations, as well as different educational tracks (scientific, literary, vocational), could provide valuable insights into the conditions that enhance or hinder the effectiveness of health literacy efforts.

A third area for future research concerns the role of emotions, stigma, and family dynamics in shaping adolescents’ perceptions of genetic risk and their openness to preventive action. Given the sensitivity of the topic and the cultural significance of marriage and reproduction in Tunisian society, educational approaches must go beyond cognitive instruction and address the emotional and relational dimensions of health literacy. Interdisciplinary research combining education sciences, medical anthropology, and psychology could help elucidate how fear, shame, family loyalty, and social expectations interact with young people’s learning processes and health behaviors.

Technological innovation also presents a promising avenue for expanding the reach and appeal of health literacy interventions. Future research should examine the integration of digital tools, such as mobile health applications, serious games, and interactive learning platforms, in the promotion of genetic awareness among youth. These tools offer opportunities for personalization, interactivity, and real-time feedback, which may be particularly effective in engaging adolescents and reinforcing learning outcomes. Evaluations of these technologies should assess not only usability and engagement but also their impact on knowledge retention, critical thinking, and behavioral intentions.

In addition, further investigation is needed into the training needs of educators tasked with delivering health-related content. Teachers play a pivotal role in mediating complex scientific information and facilitating sensitive discussions in classrooms. Research should therefore explore the competencies, attitudes, and support systems required for teachers to feel confident and prepared to engage students in topics such as thalassemia, genetic inheritance, and reproductive health. Pilot training programs and capacity-building workshops could be tested and evaluated to inform national teacher training frameworks.

Finally, it is important to pursue interdisciplinary and international collaborations that allow for knowledge exchange and comparative analysis. Lessons learned from other countries with similar epidemiological and sociocultural profiles, particularly in the Mediterranean and North African regions, can inform the development of contextually adapted strategies. Joint research projects, regional networks, and shared data platforms would strengthen the evidence base and support the formulation of coherent, evidence-informed policies for thalassemia prevention.

### Broader Public Health Implications

The insights gleaned from this study have significant implications for public health policy and practice, particularly in the context of hereditary disease prevention and health equity. Thalassemia, as a genetic disorder with profound physical, psychosocial, and economic consequences, presents a paradigmatic challenge for integrating non-communicable disease prevention within existing health frameworks. The chronic nature of thalassemia, combined with its inherited transmission and potential for stigmatization, calls for a multidimensional approach that transcends conventional biomedical interventions.

A critical implication of our findings is the recognition of health literacy as a foundational component of effective public health strategies. Enhancing genetic literacy among adolescents empowers future generations to make informed reproductive choices, reducing the incidence of new thalassemia cases and mitigating the disease burden at the population level. Such literacy is not merely the acquisition of scientific knowledge but also encompasses the development of risk awareness, emotional readiness, and social competencies necessary to navigate complex health decisions. Therefore, public health initiatives must prioritize education as a primary prevention tool alongside screening and clinical services.

Moreover, addressing thalassemia prevention through education contributes directly to the reduction of health disparities. In Tunisia and similar contexts, disparities in access to health information, healthcare infrastructure, and social support disproportionately affect marginalized populations, including those in rural or socioeconomically disadvantaged areas. By integrating genetic education into the school system, especially targeting all academic tracks and geographic regions, policy makers can promote equity in health knowledge and preventive capacities. This approach aligns with global health priorities emphasizing universal health coverage and the social determinants of health.

The psychosocial dimension of thalassemia also demands public health attention. Stigma and discrimination associated with hereditary diseases often lead to concealment, delayed diagnosis, and psychological distress for affected individuals and their families. Our study highlights the widespread misconceptions and social taboos surrounding thalassemia, which undermine both individual well-being and community support structures. Public health campaigns must therefore incorporate stigma reduction strategies, fostering inclusive attitudes and normalizing conversations about genetic risks within communities. Engaging community leaders, religious authorities, and media outlets can facilitate culturally sensitive messaging that respects prevailing values while encouraging openness.

Furthermore, the findings reinforce the imperative to adopt a life-course perspective in public health. Early intervention during adolescence, a critical developmental stage characterized by identity formation and future planning, offers a unique opportunity to shape health behaviors and attitudes that will persist into adulthood. Equipping young people with the knowledge and skills to understand and manage hereditary risks enhances their capacity for self-care and informed participation in reproductive health decisions, contributing to broader societal health gains.

Finally, the integration of thalassemia education within the public health agenda must be accompanied by systemic investments in healthcare infrastructure, including accessible genetic counseling services, diagnostic laboratories, and specialized treatment centers. Without these complementary components, educational efforts risk being undermined by gaps in service availability and quality. A comprehensive public health strategy, therefore, requires coordinated action across education, health care delivery, community engagement, and policy regulation.

In conclusion, the broader public health implications of promoting thalassemia-related health literacy are profound. This approach advances disease prevention, reduces inequities, addresses psychosocial challenges, and fosters a culture of informed health decision-making. It exemplifies the potential of health education as a transformative force within public health, particularly in contexts facing the dual burdens of hereditary and chronic diseases.

### A Call for Intersectoral Collaboration

Addressing the complex challenges posed by thalassemia prevention requires more than isolated efforts from the health or education sectors alone. It demands a concerted, intersectoral approach that brings together diverse actors across governmental, academic, civil society, and community domains. The findings of this study highlight that sustainable and effective health education interventions, particularly those targeting adolescents, must be situated within a broader ecosystem of collaboration, where shared responsibilities, resources, and expertise are mobilized in a coordinated manner.

The education sector plays a central role in this collaborative framework. Ministries of education are pivotal in integrating genetic literacy into national curricula and in ensuring that teachers are adequately trained and supported to deliver sensitive content related to hereditary diseases and reproductive health. Such efforts must be undertaken in dialogue with public health institutions, whose input ensures the scientific accuracy and relevance of educational content. Joint planning between these two sectors can foster the development of contextually appropriate modules, continuous teacher training, and systematic evaluation mechanisms to monitor outcomes over time.

The health sector, for its part, must move beyond a purely clinical approach and embrace its role in upstream prevention. Ministries of health and relevant health agencies must invest in public health communication campaigns, expand access to voluntary screening and counseling services, and facilitate the development of community-based support systems. Collaboration with schools offers a strategic entry point to reach adolescents and their families with preventive messaging and services, particularly in regions where health infrastructure may be limited. Intersectoral agreements and shared budget lines can institutionalize such cooperation and enhance its sustainability.

Academic and research institutions also have a vital role to play. Universities and teacher training institutes can contribute to the co-construction of evidence-based educational tools and to the training of a new generation of professionals who are equipped to address the intersection of genetics, education, and public health. Moreover, interdisciplinary research initiatives can generate the data needed to guide national strategies and to evaluate the long-term impact of intersectoral interventions.

Civil society organizations, including youth associations, parent groups, and patient advocacy networks, bring community knowledge, lived experience, and outreach capacity that are indispensable for grounding interventions in local realities. Their participation ensures that policies and programs are responsive to the concerns and expectations of the populations they intend to serve. They can also act as powerful mediators between institutions and communities, helping to build trust, reduce stigma, and promote active engagement in genetic health education.

Religious and cultural institutions represent another important, yet often overlooked, stakeholder. Given the centrality of marriage and family life in Tunisian society, and the influence of religious and traditional authorities on social norms, their involvement can facilitate the acceptance of preventive measures such as premarital screening and genetic counseling. Intersectoral collaboration should therefore include dialogue with these actors to ensure that public health messages are communicated in ways that are culturally respectful and socially legitimate. Ultimately, the prevention of thalassemia and the promotion of genetic health literacy must be framed as a shared societal objective. It is only through coordinated intersectoral collaboration that structural, institutional, and cultural barriers can be effectively addressed.

This study serves as a call to action for national and local decision-makers to invest in integrated policies that cut across traditional sectoral boundaries. By fostering synergies among education, health, research, civil society, and cultural institutions, Tunisia, and other countries facing similar public health challenges, can build a more resilient, equitable, and informed system of hereditary disease prevention.

### Limits

Limitations include convenience sampling and self-report biases, as noted previously. The COVID-19 pandemic further impacted reliability, with school closures reducing participation (*n* = 356/500 targeted) and remote learning limiting interviewer-student interaction. Pandemic-related stress may have influenced responses, potentially amplifying reported knowledge gaps (e.g., 35% identified thalassemia as hereditary) or stigma (e.g., 42% rural students linked it to shame), posing challenges to generalizability.

Due to the restrictions imposed by COVID-19, we opted for a convenience random sampling strategy, requiring parental consent as well as approval from school directors and teachers to conduct our study in classrooms. Despite respecting the sample size determined by the Solvin formula, the sampling was limited to two public schools in urban and rural areas, which may affect the generalizability of the results. A larger-scale longitudinal survey could confirm our findings. Additionally, our results are based on self-reported data collected by teachers and are therefore subject to social desirability bias.

### Conclusion

By situating thalassemia prevention within the broader framework of health literacy and educational equity, this study addresses a critical and underexplored gap in the literature, namely, the intersection of genetic disease prevention, adolescent education, and culturally responsive health promotion in North Africa. It demonstrates that addressing hereditary conditions such as thalassemia cannot be reduced to biomedical strategies alone; it requires cultivating an emotionally engaged and socially empowered youth population capable of making informed, autonomous, and culturally appropriate health decisions. In Tunisia, as in many Global South contexts, thalassemia prevention offers a valuable lens through which to interrogate broader questions of social justice, civic responsibility, and the transformative role of education in building equitable health systems. However, the current educational framework remains ill-equipped to meet this challenge.

Our findings show that the existing biology curriculum in Tunisian secondary schools does not foster meaningful or comprehensive understanding of genetic conditions. Students lack the conceptual and emotional tools necessary to recognize thalassemia as a serious public health issue. Misconceptions, particularly the conflation of thalassemia with iron deficiency anemia, are widespread, weakening the perceived urgency of preventive measures. Among humanities-track students, the disease is often trivialized or viewed as irrelevant, while even science-track students struggle to translate fragmented genetic knowledge into actionable understanding due to a lack of contextual and emotionally engaging learning approaches.

Despite these educational shortcomings, our data reveal a strong latent potential for empathy and prosocial motivation among Tunisian adolescents, even in the absence of formal health education. This highlights an urgent opportunity for intervention. To capitalize on this potential, we propose a stepwise roadmap for implementation, beginning with pilot programs in selected schools that integrate interactive, interdisciplinary learning modules on thalassemia and other hereditary conditions. These modules should be co-developed with local NGOs to ensure cultural relevance and contextual appropriateness. The second step involves systematic evaluation of these pilots to generate evidence for scalability. Based on this evidence, national curriculum updates can be initiated, integrating genetics and health literacy into both scientific and literary tracks. Concurrently, targeted teacher training programs must be developed to support the pedagogical shift toward participatory, emotionally resonant, and conceptually rigorous instruction.

This roadmap aligns directly with the World Health Organization’s framework for adolescent health promotion, which emphasizes the importance of health literacy, life skills education, and the active participation of youth in shaping their own health trajectories. By embedding thalassemia prevention into this broader agenda, the study not only offers a practical contribution to national curriculum reform but also advances global efforts to empower adolescents as agents of change in public health. Through this integrative approach, linking education, health, and civic engagement, Tunisia can begin to address both the immediate challenge of thalassemia and the deeper structural inequities that shape health outcomes across generations.

## Supplementary Information

Supplementary Material 1.

Supplementary Material 2.

## Data Availability

The datasets used and/or analyzed during the current study are available from the corresponding author on reasonable request.

## Declarations

### Author details

1 Laboratory of molecular and cellular hematology, Pasteur Institute of Tunis, Tunis, Tunisia.

2 ISEFC, Virtual University of Tunis, Tunis, Tunisia.

3 Faculty of Education, University of Montpellier, Montpellier, France.
