# Supplementary material for: From misconceptions to empowerment: assessing health and genetic literacy on thalassemia among Tunisian secondary school students
Source: BMC Public Health. 2025 Oct 8;25:3400. doi: 10.1186/s12889-025-24536-9 (PMC12505562; doi:10.1186/s12889-025-24536-9)
Supplement: Supplementary file 1 — Supplementary Material 1. [file 12889_2025_24536_MOESM1_ESM.docx]

**
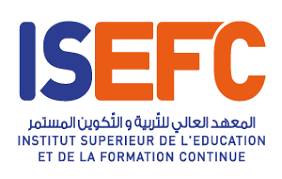

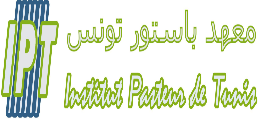
**

**HEALTH EDUCATION AND THALASSEMIA**

This investigation is coordinated by Pasteur Institute of Tunis and Higher Institute of Education and Continuing Training of Tunis. The answers to this questionnaire will enable us to study the knowledge, perceptions, attitudes and behaviors of Tunisian youth towards thalassemia. Thank you for your participation.

**General information**

**Gender**: Male Female

**Age**: ……………………………

**School name**: …………………………… **Region**: ……………………………

**School level/specialty**:

3rd year: Sciences Mathematics Letters Techniques Economics

4th year: Sciences Mathematics Letters Techniques Economics

**Q1. A.** **Thalassemia is illnesses:** (*Check one box only*)

1. infectious 2. Hereditary 3. dietary diseases 4. I don't know

**Q2. B. Thalassemia has the following symptom(s):** (*Check one or more boxes*)

1. Skin rashes
2. Fever
3. Severe anemia
4. Dry cough and sore throat
5. Jaundice (icterus)
6. Limb pain (feet, hands, etc.)
7. Blood in urine
8. I don't know

**Q3. C. Thalassemic patients are prescribed the following treatment(s):** (*Check one or more boxes*)

1. Antibiotics
2. Food supplements (vitamins, minerals, etc.)
3. Blood transfusions
4. Iron
5. I don't know

**Q4. D. What laboratory test is used to identify a hemoglobin disorder?** (*Check one box only*)

1. Urine test
2. Electrophoresis test
3. Blood count
4. Induced hyperglycemia
5. Lipid balance
6. I don't know

**Q5. E. What is your main source of information about blood diseases?** (*Check one box only*)

1. TV, radio, newspapers
2. Internet search engines (google, etc.)
3. Social networks (Facebook, X, Instagram, etc.)
4. Life Sciences textbooks (School, College, etc.)
5. Healthcare professionals (nurses, doctors, pharmacists, etc.)
6. Your entourage (family, friends, etc.)
7. Health clubs ( Secondary school)
8. Nothing

**Q6. F. According to its source, evaluate the information utility on** **thalassemia.** (*Check one box only*)

| **Information sources** | **Not useful** | **Useful** | **Very useful** |
| --- | --- | --- | --- |
| 1. National media (TV, radio, etc.) |  |  |  |
| 2. Search engines (google, etc.) |  |  |  |
| 3. Social media (Facebook, X, Instagram, etc.) |  |  |  |
| 4. Life Sciences curriculum |  |  |  |

**Q7. G1. Rate your "thalassemia" fear on an increasing scale from 1 to 7** *(Check one box only)***.**

**Not afraid**

**Very afraid**

**Q9. I'm now going to propose some opinions on thalassemia.** *Tell me, for each of them, whether you totally agree, somewhat agree, somewhat disagree or totally disagree.*

| **Opinions** | **Totally agree** | **Somewhat agree** | **Somewhat disagree** | **Totally Disagree** |
| --- | --- | --- | --- | --- |
| **H.** Some thalassemia forms are contagious. |  |  |  |  |
| **I.** When you have thalassemia, it's better to hide it from those around you (friends, etc.) to live a normal life. |  |  |  |  |
| **J.** When you have thalassemia, you won't be able to study or work like the others. |  |  |  |  |
| **K.** If you have thalassemia, you must inform your partner of your illness before getting married. |  |  |  |  |
| **L1.** We must help children with thalassemia. (Join an association, donate money, etc.). |  |  |  |  |
| **L2.** We must donate blood, the sick need it. |  |  |  |  |

**Q10. For each of the following items,** *check only one box "Yes", "No", "No idea".*

| **Questions** | **Yes** | **No** | **No idea** |
| --- | --- | --- | --- |
| **M.** Can you "catch" thalassemia like the flu? |  |  |  |
| **N.** Can a man with thalassemia avoid having sick children? |  |  |  |
| **O.** Can a child carrying a thalassemia allele (heterozygote) develop symptomatic thalassemia? |  |  |  |
| **P.** Could a husband with homozygous thalassemia and his healthy (non-carrier) wife have a boy with symptomatic thalassemia? |  |  |  |
| **Q.** Can a woman with thalassemia avoid having sick children? |  |  |  |
| **R.** Could a husband who is heterozygous for thalassemia and his wife who is homozygous for thalassemia have a daughter suffering from the disease? |  |  |  |

**Q11. Faced with the idea of transmitting thalassemia to your children, what do you think of prenatal diagnosis or genetic counseling for each of the following cases?** *(Check one box only)*

| **Situations / Diagnosis / Genetic counseling** | **Not useful** | **Useful** | **No idea** |
| --- | --- | --- | --- |
| **S.** Premarital genetic counseling when one partner is ill. |  |  |  |
| **T.** Premarital genetic counseling when both partners are ill. |  |  |  |
| **U.** Prenatal diagnosis when the couple is healthy, but the husband has the disease in his family. |  |  |  |
| **V.** Prenatal diagnosis when the couple is healthy, but the woman has the disease in her family. |  |  |  |

**Q12. Evaluate the risk of having children with symptomatic thalassemia in the following situations:** (Check one box only)

| **Situations** | **No Risk** | **Obvious risk** | **No idea** |
| --- | --- | --- | --- |
| **W.** A couple who both carry the disease allele (heterozygotes). |  |  |  |
| **X.** A couple, one of whom is a carrier allele for the disease (heterozygous). |  |  |  |
| **Y.** A couple where only the wife has the disease. |  |  |  |
| **Z.** A couple where only the man has the disease. |  |  |  |
