# Supplementary material for: From misconceptions to empowerment: assessing health and genetic literacy on thalassemia among Tunisian secondary school students
Source: BMC Public Health. 2025 Oct 8;25:3400. doi: 10.1186/s12889-025-24536-9 (PMC12505562; doi:10.1186/s12889-025-24536-9)
Supplement: Supplementary file 2 — Supplementary Material 2. [file 12889_2025_24536_MOESM2_ESM.docx]

| **HL** | **Access and obtain thalassemia information** | **Understanding thalassemia information** | **Evaluation thalassemia information** | **Application and use thalassemia information** |
| --- | --- | --- | --- | --- |
| **Thalassemia care** | 1) Ability to access information on medical or clinical problems | 2) Ability to understand medical information and derive meaning | 3) Ability to interpret and evaluate medical information | 4) Ability to make informed decisions on medical issues |
| **Items** | **E, F** | **A, B, C, D / G1** | **A, B, C, D** | **C, D** |
| **Thalassemia prevention** | 5) Ability to access thalassemia information (incidence, severity, symptoms, diagnosis, etc.) | 6) Ability to understand and derive meaning from thalassemia information and its transmission mode | 7) Ability to interpret and assess transmission risk of thalassemia in real-life situations | 8) Ability to make informed decisions about procreation (partner information, screening, MAP, etc.). |
| **Items** | **E, F** | **A, B, C, D / G1** | **H, I, J, K, M, N, 0, P, Q, R / W, X, Y, Z** | **I, K, S, T, U, V** |
| **Health promotion**  **Applied to thalassemia** | 9) Ability to keep abreast of health determinants in the social and physical environment (stigmatization, difficult access to care, blood, medication, etc.). | 10) Ability to understand information on health determinants in the social and physical environment (stigmatization, difficult access to care, blood, medication, etc.) and derive meaning from it. | 11) Ability to interpret and evaluate information on health determinants in the social and physical environment (stigmatization, difficult access to care, blood, medication, etc.). | 12) Ability to make informed decisions about the health determinants in the social and physical environment (school activities, blood donation, joining an association, etc.). |
| **Items** | **E, F** | **A, B, C, D / G1** | **I, J** | **L1, L2** |
| **HL typology** | **Interactive literacy** | **Functional literacy** | **Critical literacy** | **Critical literacy** |

***Appendix 1: Matching the thalassemia items to the four HL dimensions and the three health domains***
